# Ultrashort spin–orbit torque generated by femtosecond laser pulses

**DOI:** 10.1038/s41598-022-24808-z

**Published:** 2022-12-13

**Authors:** T. Janda, T. Ostatnický, P. Němec, E. Schmoranzerová, R. Campion, V. Hills, V. Novák, Z. Šobáň, J. Wunderlich

**Affiliations:** 1 Institute for Experimental and Applied Physics, University of Regensburg, Universitätsstr. 31, 93053 Regensburg, Germany; 2 Faculty of Mathematics and Physics, Charles University, Ke Karlovu 3, 121 16 Prague 2, Czech Republic; 3 School of Physics and Astronomy, University of Nottingham, Nottingham, NG7 2RD UK; 4 Institute of Physics ASCR, v.v.i., Cukrovarnická 10, 162 00 Prague 6, Czech Republic

**Keywords:** Electronic devices, Optoelectronic devices and components, Electronic and spintronic devices, Spintronics

## Abstract

To realize the very objective of spintronics, namely the development of ultra-high frequency and energy-efficient electronic devices, an ultrafast and scalable approach to switch magnetic bits is required. Magnetization switching with spin currents generated by the spin–orbit interaction at ferromagnetic/non-magnetic interfaces is one of such scalable approaches, where the ultimate switching speed is limited by the Larmor precession frequency. Understanding the magnetization precession dynamics induced by spin–orbit torques (SOTs) is therefore of great importance. Here we demonstrate generation of ultrashort SOT pulses that excite Larmor precession at an epitaxial Fe/GaAs interface by converting femtosecond laser pulses into high-amplitude current pulses in an electrically biased *p*-*i*-*n* photodiode. We control the polarity, amplitude, and duration of the current pulses and, most importantly, also their propagation direction with respect to the crystal orientation. The SOT origin of the excited Larmor precession was revealed by a detailed analysis of the precession phase and amplitude at different experimental conditions.

## Introduction

The ever-increasing demand for a faster and low-energy-consumption electronics calls for a development of electronic devices with high operational frequencies. Spintronics is among the most frequently considered solutions, and currently represents a highly active and rapidly developing field placed at the intersection of relativistic quantum physics, materials science, and nanoelectronics. Commercial spin-based devices such as hard disk drives and magnetic random-access memories are intrinsically robust against charge perturbations and non-volatile. These applications rely on two opposite magnetisation orientations representing logic “zero” and “one” and the fastest speed of switching magnetization between the two orientations is limited by the Larmor precession. Magnetization reversal has been realized by transferring linear momentum into spin angular momentum through the spin Hall effect (SHE) and the inverse spin galvanic effect (iSGE), and the underlying spin–orbit fields have been studied extensively in ferromagnetic (FM)/non-magnetic (NM) systems. In these bilayer systems, a spin current generated in the NM bulk or at the FM/NM interface is absorbed in the adjacent FM layer, and the corresponding spin–orbit fields are, therefore, primarily interfacial effects^1–5^. Epitaxial interfaces are of particular interest, since effective spin–orbit fields may depend on the current direction with respect to crystallographic orientation^6–8^.

In this work, we demonstrate the excitation of Larmor precession at an epitaxial iron-gallium arsenide (Fe/GaAs) interface by ultrashort (sub-picosecond) spin–orbit torque (SOT) pulses. We use a magneto-photo-electric device, where the Fe is epitaxially grown on a silicon-doped n-GaAs/intrinsic GaAs/carbon-doped p-GaAs photodiode structure. The metal/semiconductor interface is forming a Schottky junction in series with the *p*-*i*-*n* photodiode. Irradiation of the electrically biased Fe/*n*-*i*-*p*-GaAs heterostructure with femtosecond laser pulses can generate ultrashort sub-ps current pulses with up to ~ 100 mA large amplitudes that propagate along the GaAs/Fe interface. The magnitude and polarity of the applied bias voltage allow to control the amplitude and polarity as well as the duration of the current pulses. In addition, as it is particularly important for this study, we can also control the lateral propagation direction of the current pulses with respect to both the GaAs crystal orientation and the magnetic easy axes of the Fe film. We realize this by focusing the excitation laser pulse to a specific position in our disk-shaped device. By carefully analyzing phase and amplitude of the induced Larmor precession we identify conditions where its excitation is dominated by SOT pulses.

## Results

### Studied sample

**Figure 1:**
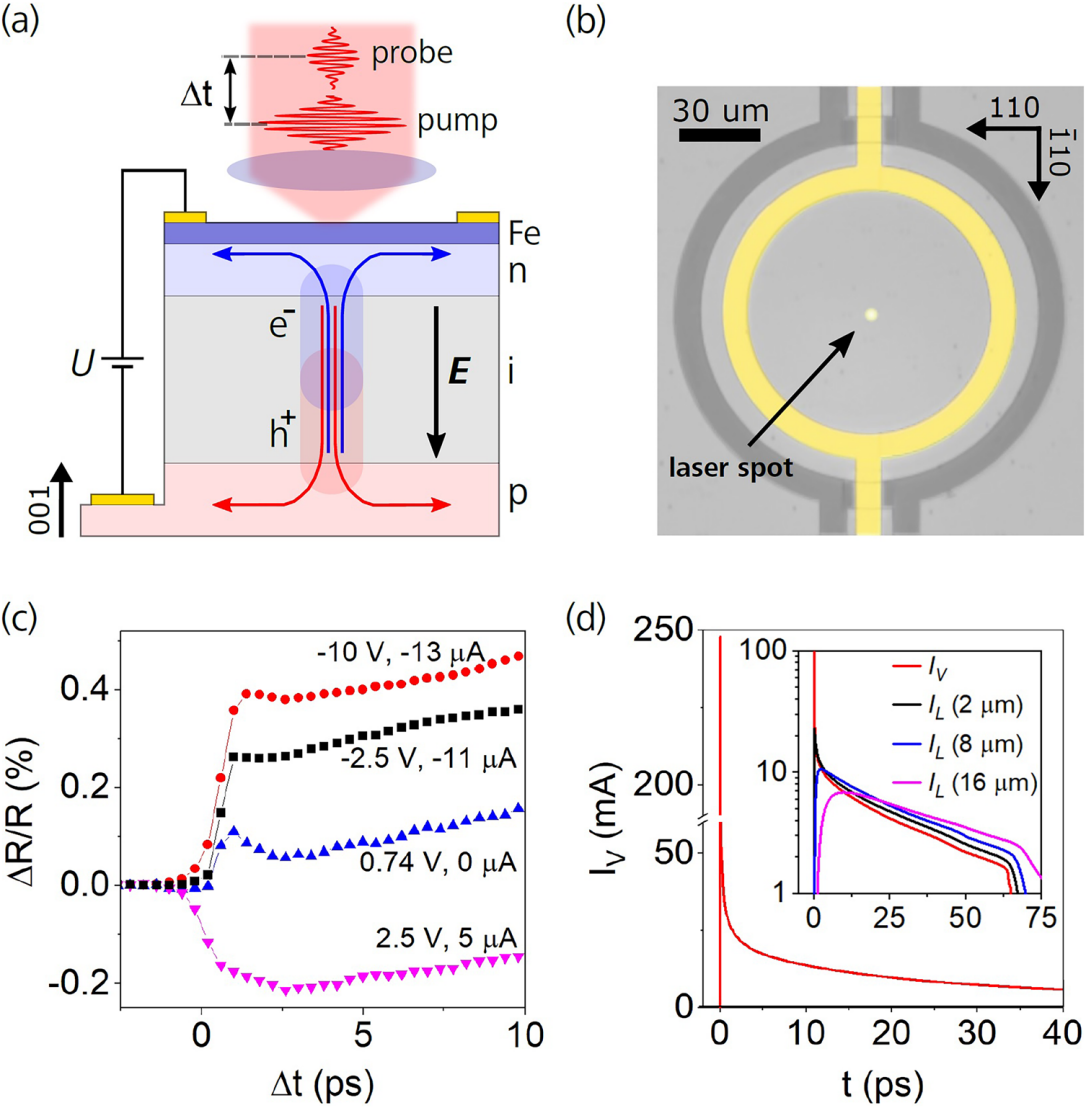
Device structure and properties of the photogenerated current pulses. (**a**) Schematic illustration of the GaAs *p-i-n*-diode structure with a thin ferromagnetic Fe film deposited directly on top of the diode. After an impact of a femtosecond laser pulse, a cloud of electron–hole pairs is generated. The electrons (*e*^−^) and holes (*h*^+^) are separated by a strong electric field ***E*** in the intrinsic layer (*i*) and accelerated towards the oppositely-charged electrodes creating a short current pulse, whose direction is schematically shown by arrows. To measure the dynamics of excited charge, spin and magnetization, a second time-delayed laser pulse can be used. **(b)** Micrograph of the photodiode lateral structure. The studied Fe/GaAs diode (light grey) is isolated from the rest of the sample by a trench (dark grey). The Fe film is electrically contacted by Au circular contact (yellow), which bridges the trench. The Au contact to the bottom p-GaAs electrode is outside of the displayed image. The bright spot in the image center is the focused laser spot. **(c)** Transient reflectivity measured in a pump-probe experiment for different applied bias voltages indicating the sub-picosecond photocurrent onset. The measured photocurrent averaged over the period of the stroboscopic experiment is shown for each bias. **(d)** Numerical simulation of the vertically propagating photocurrent pulse entering the *n*-GaAs/Fe electrode for an external bias of − 10 V. The initial current spike decays at a timescale comparable to the laser pulse duration of ≈ 100 fs. Inset: Comparison of the vertical current (*I*_*V*_) and the lateral current (*I*_*L*_) captured at various distances (2, 8, and 16 µm) from the injection point illustrating the pulse dispersion during its propagation through the device; note that logarithmic *y*-scale is used in the Inset.

In Fig. 1a we show a sketch of our GaAs *p*-*i*-*n*-diode grown on a semi-insulating GaAs substrate. On top of the diode (on the *n*-doped layer) an ultrathin (2 nm ≈ 14 monolayers) ferromagnetic Fe film is deposited and protected by an aluminum-oxide layer (see Supplementary Fig. S.3 and Note 3 for details on magnetic properties of the studied sample). The GaAs diode consists of a 670 nm thick *p*-doped layer (carbon-doped, *n*_*C*_ = 2 × 10^18^ cm^−3^), a 1000 nm thick intrinsic layer, and three *n*-doped layers with a silicon dopant concentration gradually increasing towards the diode surface: 150 nm thick layer with *n*_*Si*_ = 1 × 10^17^ cm^−3^, 15 nm thick layer with *n*_*Si*_ ranging from 1 × 10^17^ to 5 × 10^18^ cm^−3^, and 15 nm thick layer with *n*_*Si*_ = 5 × 10^18^ cm^−3^. The *p*-*i*-*n*/Fe stack was patterned into a disc with a diameter of 100 µm, as shown in the micrograph in Fig. 1b. Here, the circularly shaped 100 nm thick Au-contact (yellow) is used to electrically connect the thin Fe film (light grey), which is separated from the rest of the layer by a trench (dark grey). The built-in electric field *E*, which is present in the intrinsic layer of the diode, is superimposed by an additional field generated by an applied bias voltage between the two Au contacts connecting both the *p*-doped GaAs and the Fe film on top. Note that the forward (reverse) voltage is positive (negative) in our convention. As shown in Supplementary Fig. S.4a, without laser irradiation the diode can withstand a large reverse bias voltage with negligible leakage current. Moreover, we also can apply a relatively large forward voltage of up to + 8 V without incurring large dark currents of more than 200 μA due to the formation of an additional rectifying Schottky barrier at the Fe/*n*-GaAs interface^9^. In summary, the currents without laser irradiation are at least 3 orders of magnitude smaller than the amplitudes of the current pulses excited by fs laser pulses over the entire bias voltage range applied in our experiments.

### Temporal characteristics of the photocurrent pulse

In order to produce ultrashort electrical current pulses of large amplitudes, the electrically biased *p*-*i*-*n*/Fe structure is illuminated by a focused femtosecond laser beam. Absorption of the laser pulse in the µm-thick intrinsic layer creates electron–hole pairs which are instantaneously separated by the large internal electric field and accelerated towards the opposite electrodes creating an ultrashort current pulse, as schematically shown in Fig. 1a. Two stroboscopic time-resolved techniques were applied to explore the temporal characteristics of the current pulses generated in our photodiode. Pump-probe reflectivity measurements were used to identify the sub-picosecond onset of the current pulses. Photocurrent correlation measurements were used to monitor the time characteristics of the decaying tail of the pulses on the scale of tens of picoseconds. These two techniques, therefore, provide complementary information about the electrical pulses. For more information see Methods section.

Figure 1c shows differential reflectivity data measured in the pump-probe experiment, i.e., evolution of the sample reflectivity triggered by an impact of the pump laser pulse at time delay Δ*t* = 0. The pump and probe laser spots with a diameter of ≈ 1 µm were overlapped in the center of the device. Differential reflectivity, which is connected with the transient photo-generated charge carriers, has been previously used to estimate the electrical pulse duration^9^. Here it provides information about the onset of the electrical pulse. To interpret the measured differential reflectivity data, we first consider the case where the built-in electric field of the *p*-*i*-*n* diode is almost compensated by an applied voltage bias causing *zero* averaged photocurrent. In this case, the transient reflectivity varies on the fast-scale only by a small amount. With a *non-zero* averaged photocurrent and a corresponding non-zero internal electric field, the onset of the transient reflectivity variation increases with increasing magnitude of the applied bias and the sign of the variation switches when the polarity of the photocurrent changes [see Fig. 1c]. Most importantly, the reflectivity changes within a sub-picosecond range. We attribute the bias-dependent reflectivity variation to the Franz-Keldysh effect^10,11^, as the photoexcited charge carriers separate from each other very quickly and screen the internal electric field accordingly. The fast rising and falling current until the screening has been established corresponds to an ultrafast initial current spike. Its duration is, therefore, equal to the time span of the measured reflectivity variation.

The sub-picosecond time scale of the current spikes identified by the transient reflectivity measurements is also confirmed by theoretical modeling, see Fig. 1d. The simulation shows that the photoexcited electrons and holes in the intrinsic region of the diode are accelerated by the strong internal electric field towards the opposite electrodes thus causing an immediate onset of the photocurrent (see Supplementary Note 6 for details). As the two oppositely charged carrier clouds start to separate, the associated electric field pointing against the externally applied bias arises at a sub-picosecond timescale causing the rapid sub-picosecond decay of the current. The corresponding current spikes reach amplitudes of hundreds of mA. As apparent in Fig. 1d, following the initial sub-ps current spike there is a further slower decay of the flowing current, which proceeds at a timescale of tens of picoseconds. It results from a drainage of the remaining photo-charges and a restoration of the internal strong electric field and it corresponds to the current pulse tail. In Fig. 1d we show the simulated current pulse flowing vertically (i.e., perpendicularly to the sample plane) in the micrometer-wide laser-pulse-illuminated channel at − 10 V. As shown in Fig. S.6a, the current pulse tail becomes longer for smaller biases and it is terminated when all the photogenerated charge is drained away from the illuminated spot. A detailed description of the performed numerical simulations can be found in Supplementary Notes 6 and 7.

The decay of the current pulse tails in the presence of the screening electric field was experimentally studied by the photocurrent correlation measurements for various applied biases (see Supplementary Fig. S.5 and Note 5). For ≈ 1 µm laser spot size, decay times ranging from 20 ps at − 10 V applied bias to ≈ 175 ps at zero applied bias, where the photocarriers are accelerated only by the built-in field at the *p*-*i*-*n* junction, were measured [see Supplementary Fig. S.5a and Fig. 4d]. For larger laser spots, the decay times become longer due to the presence of electric screening in the wider photoexcited area [see Supplementary Fig. S.5b]. Comparing the Larmor frequencies of the thin Fe layer, which are tens of GHz [see Fig. S.3b], with the temporal characteristics of the current pulses generated by femtosecond laser pulses in our device, we conclude that the observed Larmor precession is excited mainly during the 10-picosecond tail of the current pulse. Nevertheless, the sub-picosecond current spike, with its high current amplitude, could become relevant for excitation of antiferromagnetically ordered materials where the precession frequencies reach the THz scale^12–14^.

As illustrated schematically in Fig. 1a, after reaching the Fe/n-GaAs electrode the electron current pulse flows in lateral direction. In the inset of Fig. 1d, we plot the vertical current pulse generated in the center of our disc-shaped device together with the lateral current pulses captured at different distances from the disc center to illustrate the evolution of the current pulse during its propagation through the device. To allow for a direct comparison to the vertical current pulse, the lateral surface current densities obtained by the simulation [see Fig. S.6c] were integrated along the circumference of *2πr* for each individual distance *r* from the device center.

### Magnetization dynamics induced by spin–orbit torque pulses

**Figure 2:**
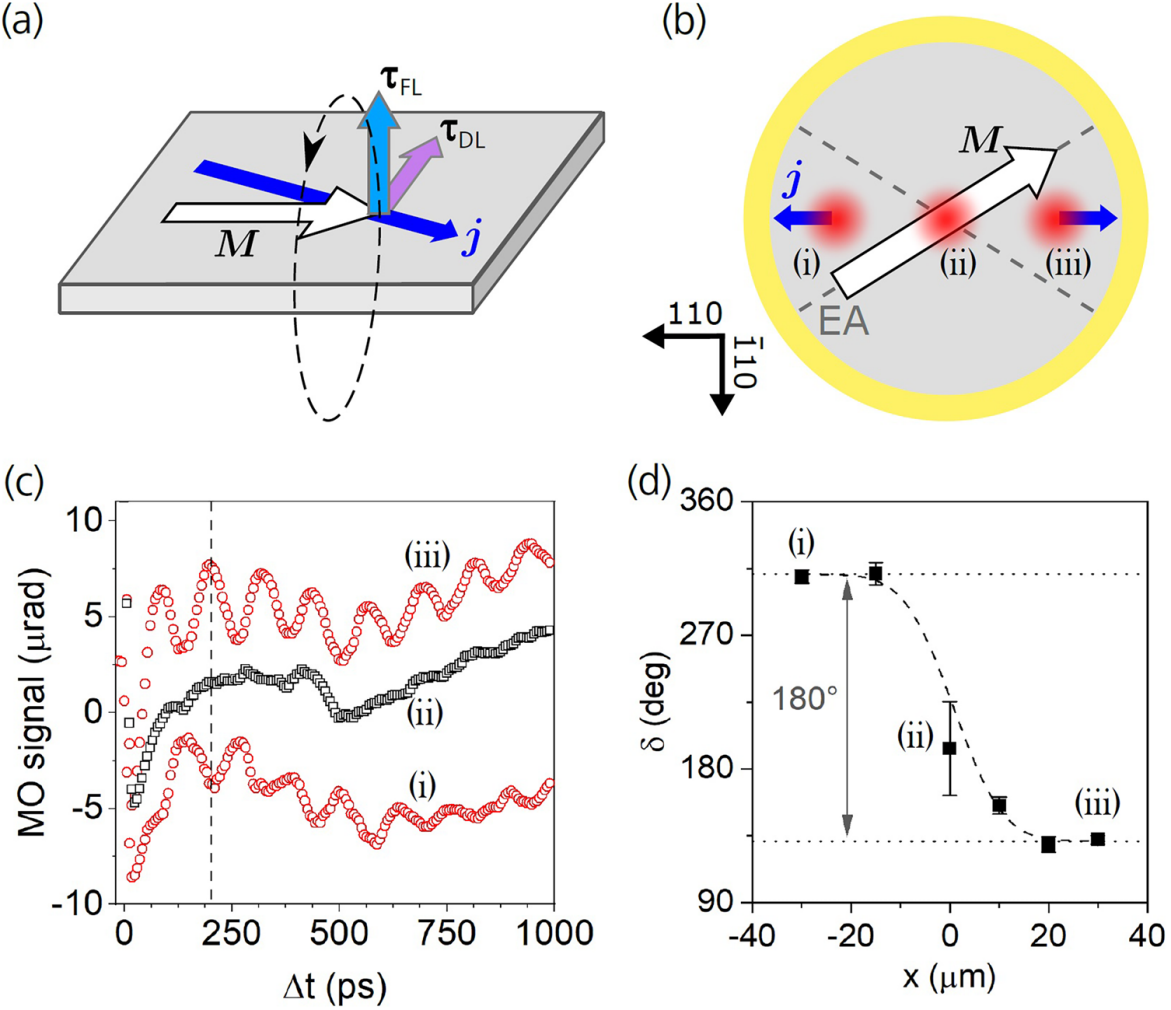
Magnetization precession induced by ultrashort SOT pulse. (**a**) Schematic illustration of the two possible SOT contributions acting on magnetization (***M***) due to the photocurrent pulse (***j***): field-like (***τ***_*FL*_) and damping-like (***τ***_*DL*_) torques. The two orthogonal torques lead to magnetization precessions (dashed ellipse) that differ by 90° in the initial phase. **(b)** Sketch of the photodiode with three positions located in the device center and ± 30 µm away from the center along the [110] crystallographic direction, which were investigated by the spatially-overlapped pump and probe laser spots. The net photocurrents in positions (i) and (iii) are opposite due to the location of the annular Au contact. No net lateral photocurrent is generated in position (ii). The experiment was performed with no external magnetic field applied when ***M*** was oriented along one of the easy axes (EA), which are indicated by the dashed lines. **(c)** Magnetization precession measured in the three positions indicated in (**b**) for applied bias of − 10 V; the curves are vertically shifted for clarity. The precessional signal is correlated with the polarity and magnitude of the lateral current pulse. **(d)** Points: initial precessional phase *δ* as a function of the laser spots position *x* within the photodiode. The value *δ* ≈ 135° for *x* > 0 indicates the presence of damping-like SOT, as discussed in the text. Dashed line: fit by a step-like function smeared out by the Gaussian laser beam profile with ≈ 25 µm diameter.

To study the magnetization dynamics induced by spin–orbit torque pulses, we need to consider not only the temporal properties of the generated current pulse, but also its propagation direction. Due to our vertical photodiode design, the photocurrent pulses can be generated locally at any position within the disk-shaped pillar structure by positioning the focused laser spot inside the annular Au contact. As illustrated in Fig. 2b for 3 different spot positions, the net flux of the laterally propagating current pulse is always directed towards the shortest distance to the annular Au contact providing a unique and simple opportunity to control the orientation of the lateral current pulses in only one single device.

As described in detail in Supplementary Note 6, the photocurrent pulse generated in the device initially flows in the vertical direction and after reaching the top Fe/*n*-GaAs electrode it propagates laterally toward the annular Au contact. To eliminate the effect of the Oersted magnetic field generated by the vertically propagating current pulse, which was investigated in^9^, we performed experiments with pump and probe laser spots focused at the same position. To achieve a precise spatial overlap of pump and probe beams, we used relatively wide laser spots with a diameter of about 25 µm (see “Methods” section for more details). The radially symmetric distribution of the Oersted field generated by the vertical current in the pump-illuminated area averages out to zero within the spatially overlapped probing area and, therefore, a possible magnetization precession arises only from torques generated by the lateral current.

The lateral current pulse can act on the iron magnetization again via the associated Oersted magnetic field, which is a *non-local* effect. Therefore, the field generated by the electron current flowing in the top *n*-doped GaAs layer is compensated to a large extent by the hole current flowing in the bottom *p*-doped GaAs layer as both types of charge carriers move in the same direction coupled by the Coulomb interaction (see Supplementary Note 6). On the other hand, the spin–orbit torque is a *local* effect originating from the Fe/*n*-GaAs interface and, therefore, it is generated only by the electron current. The part of the lateral charge current flowing through the *n*-GaAs layer can also contribute to SOT via the spin Hall effect (SHE). However, this contribution is expected to be negligible because of the very small spin Hall angle in *n*-GaAs of 6 × 10^–3^ at low temperatures^15^ and because of the very large conductivity difference of ~ 10^3^ between the metallic Fe film and the *n*-GaAs, which makes most of the lateral current flowing through the Fe film. The negligible role of SHE in our measurements was confirmed in a test experiment in which spin-polarized photocurrent pulses were generated in GaAs via optical orientation by circularly polarized pump laser pulses^16^; see Supplementary Note 2. Despite the high degree of the photo-electron spin polarization in GaAs of up to 50% which was oriented perpendicular to the iron magnetization, no evidence of transfer of the spin polarization from the bulk GaAs to the iron magnetization was observed. A plausible explanation is a reduction of efficiency of the spin injection through the metal/semiconductor interface due to the presence of a Schottky barrier^17^. Hence, we conclude that the much weaker contribution to the torque on the iron magnetization due to the spin current generated by the SHE in *n*-GaAs is negligible. In summary, a possible magnetization precession observed in the configuration with spatially overlapped pump and probe laser spots can only be induced by an effective spin–orbit field generated at the Fe/*n*-GaAs interface by the lateral photocurrent pulse. Other current pulse-unrelated effects that could in principle excite magnetization precession can be excluded in our experiments, as discussed in detail in Supplementary Note 1.

**Figure 3:**
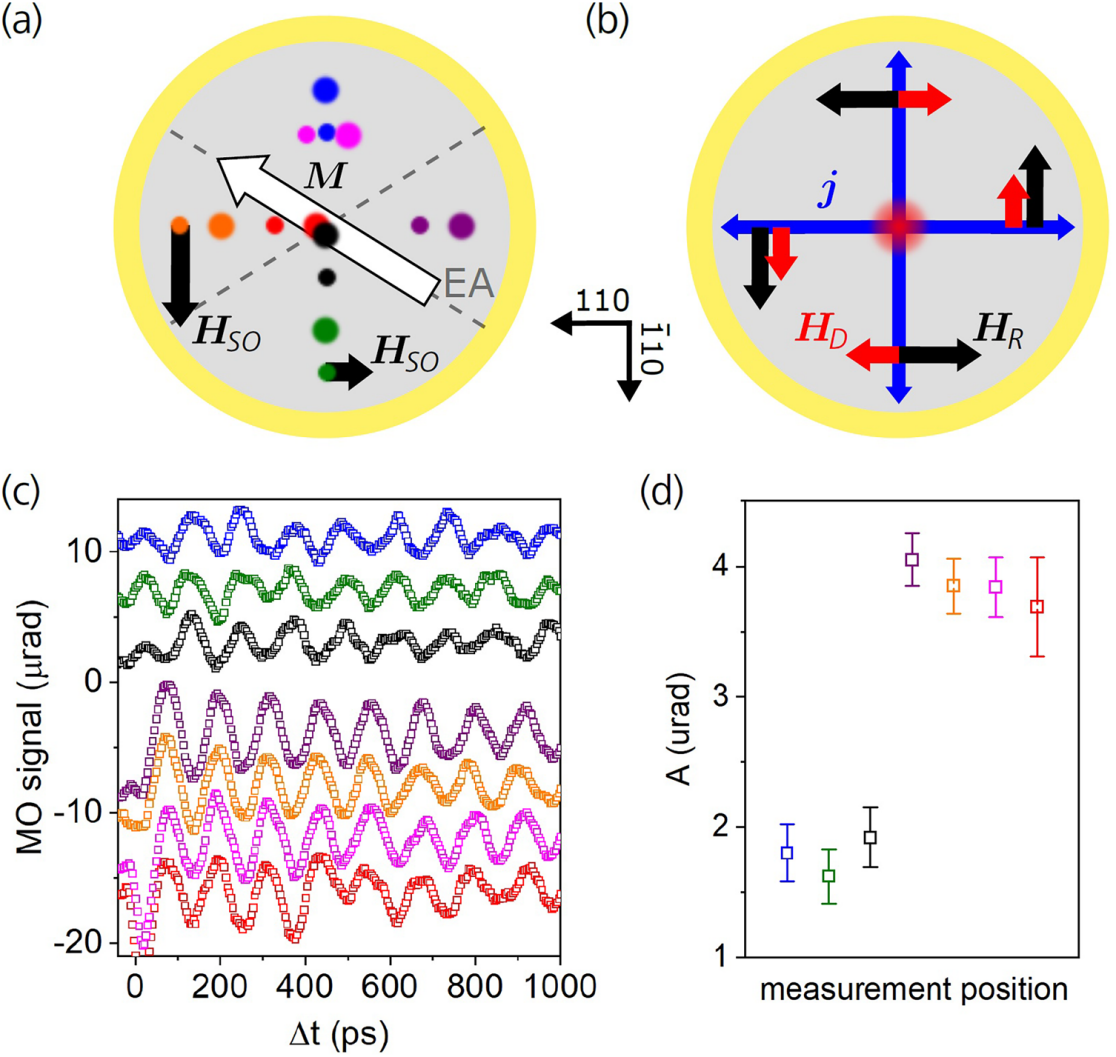
Anisotropy of the SOT-induced precession amplitude with respect to the photocurrent direction. (**a**) Schematic illustration of the photodiode with positions of spatially-separated pump and probe laser spots indicated by different colors. The larger and smaller dots indicate the pump and probe spot positions, respectively (with a diameter of ≈ 1 µm), which were always 9 µm apart with the probe spot displaced either along [$\overline{1}10$] or [$110$] crystallographic direction. The directions of the magnetization ***M*** (white arrow) and the local total spin–orbit field ***H***_*SO*_ (black arrows) are also indicated. **(b)** Directions of the Bychkov-Rashba (***H***_*R*_, black arrows) and Dresselhaus (***H***_*D*_, red arrows) contributions to the total spin–orbit field ***H***_*SO*_ for different photocurrent directions (blue arrows) in the device. Combination of the two anisotropic contributions results in different magnitudes of ***H***_*SO*_ when probing along [$\overline{1}10$] and [$110$] in (**a**). **(c)** Magnetization precession measured for applied bias of − 10 V at positions indicated in (**a**) by the corresponding colors; the curves are vertically shifted for clarity. **(d)** Precessional amplitude *A* extracted by fitting the data in part (**c**) by Eq. (1) for Δ*t* > 100 ps. The colors of the data points correspond to that used in parts (**a**,**c**). The larger magnetization precession amplitude observed for current pulses propagating along [110] direction is in agreement with the stronger ***H***_*SO*_ compared to the case of pulses propagating along [$\overline{1}10$] direction.

The spin–orbit field arises from a broken inversion symmetry both due to the epitaxial Fe/GaAs interface and due to the missing inversion centre of the zinc-blende crystal structure of the GaAs bulk^18,19^. The respective contributions, the Bychkov-Rashba-like field ${\varvec{H}}_{R} \sim\left( {k_{x} {\varvec{e}}_{y} - k_{y} {\varvec{e}}_{x} } \right)$ and the Dresselhaus-like field ${\varvec{H}}_{D} \sim\left( {k_{y} {\varvec{e}}_{y} - k_{x} {\varvec{e}}_{x} } \right)$, lie in the plane of the Fe/GaAs interface and they depend linearly on the lateral components of the electron linear momentum $\hbar {\varvec{k}}$^8^; see also Fig. 3b. Here, ${\varvec{e}}_{x}$ and ${\varvec{e}}_{y}$ are unit vectors pointing along [100] and [010] crystallographic directions of our epitaxially grown Fe/GaAs structure, respectively. Hence, when a lateral charge current is applied, Bychkov-Rashba- and Dresselhaus-like fields superimpose to an effective spin–orbit field which scales linearly with the current magnitude and whose magnitude and orientation also depend on the current direction; see Fig. 3a. Depending on whether the spin lifetime in iron is shorter or longer than the spin precession time in the exchange field of iron, the Bychkov-Rashba- and Dresselhaus-like spin–orbit fields can act both as field-like SOT ($\tau_{FL}$) and damping-like SOT ($\tau_{DL}$), respectively. Both timescales are of the order of tens of femtoseconds, i.e., much shorter than the duration of our picosecond photocurrent pulses. We therefore consider the onset of the SOT to be immediate with respect to the duration of our photocurrent pulses. The field-like and damping-like SOTs act on the magnetization in two orthogonal directions tilting the magnetization perpendicular and parallel to the sample plane, respectively, as depicted in Fig. 2a. Accordingly, current pulse-induced magnetization precessions caused by the damping-like SOT only and by the field-like SOT only would be mutually phase shifted by 90°.

The direction of the lateral current pulse flowing in our photodiode from the pump laser spot towards the annular Au contact depends on the laser spot position, as depicted in Fig. 2b. When the laser spot is focused in the center of the disc, the angular distribution of the lateral current flowing towards the ring-shaped contact is equal in all directions. Therefore, within the spatially overlapped probe laser spot, the net lateral current equals to zero and, consequently, no net spin–orbit torque is expected. In contrast, when the overlapped laser spots are moved out of the device center, the lateral current distribution becomes asymmetric and the current density is the largest in the direction of the spot-displacement since here the distance to the annular Au contact is the shortest. The corresponding net current flow directions are indicated in Fig. 2b by blue arrows.

In Fig. 2c we show magneto-optical (MO) pump-probe traces measured at three different positions indicated in Fig. 2b. The measured dynamic MO signal is due to polar MO Kerr effect which is proportional to the perpendicular-to-plane magnetization component (see Supplementary Note 1 for details). While the precessional amplitude is almost zero at the disc center, Larmor precession of magnetization is clearly apparent when the spatially-overlapped pump and probe laser spots are displaced by ± 30 μm away from the disc center along the $\left[ {110} \right]$ and $\left[ {\overline{11} 0} \right]$ directions. Importantly, these precessions are mutually phase-shifted by 180$^\circ$, as indicated by a vertical dashed line in Fig. 2c. The presence (absence) of the magnetization precession and its opposite phases are clearly correlated with the presence (absence) and opposite directions of the lateral current pulses at the 3 different positions. This observation confirms that the precession is triggered by the lateral current pulses and it excludes any thermally-induced effects, such as temperature-induced magnetic anisotropy variations (see Supplementary Note 1 for a detailed discussion).

The measured pump-probe traces were fitted by a damped harmonic function

1 $$MO\left( {{\Delta t}} \right) = { }A{\text{ cos}}\left( {2{\uppi }f{\Delta }t + \delta } \right)e^{{ - \frac{{{\Delta }t}}{{\tau_{d} }}}} { } + P_{4} ,$$

where *A*, *f*, *δ*, and *τ*_*d*_ are precessional amplitude, frequency, initial phase and damping time, respectively. *P*_*4*_ is a 4th-order polynomial used to remove the signal background which is not related to the magnetization precession. In Fig. 2d we show the initial phase of the magnetization precession measured at different positions along the [110] crystallographic direction. As already mentioned, the phase changes by 180° when crossing the device center, i.e., when the current pulse propagation direction is inverted. The width of the transition region corresponds to the used laser beam size, which is ≈ 25 μm in this particular experiment. Importantly, the experimentally measured value of the initial phase *δ* is ≈ 135° (and 315°) for a given net-current direction. As we discuss in Supplementary Note 1, the initial phase of the magnetization precession depends not only on the direction of the corresponding torque, but also on the duration of the stimulus. In the measurements shown in Fig. 2, where the current pulse duration [see Supplementary Fig. S.5b] is significantly longer than the Larmor precession period of ≈ 100 ps [see Supplementary Fig. S.3b], a phase of ≈ 90° is expected for a precession excited by the current pulse-induced Oersted field. This effect, therefore, can’t explain the observed phase of ≈ 135°, in agreement with the aforementioned strong suppression of the Oersted field effects in our experimental geometry. On the other hand, the measured precession phase can be explained by a superposition of the field-like and damping-like SOTs which would lead to precession phases of ≈ 90° and ≈ 180°, respectively.

To further investigate the importance of SOT relative to the Oersted-field torque for inducing the magnetization precession, we performed additional MO pump-probe experiments in a configuration with pump and probe laser spots being spatially separated. In this case, both SOT from the lateral current pulse and the Oersted-field torque from the vertical current pulse are present. The net direction of the lateral current pulse propagation within the probed area is determined by the position of the probe laser spot relative to the pump spot, which is the origin of the laterally propagating current pulse. To achieve the required spatial separation between the pump and probe laser spots within the device, we used rather small laser spot size of only ≈ 1 μm for this set of experiments. The tightly focused pump laser pulse excitation produced much shorter current pulses than in the previously described experiments with ≈ 25 µm wide laser spots, which is because of a reduced effect of charge-screening in the case of smaller spots [cf. Supplementary Fig. S.5a,b]. We first investigated the dependence of the induced Larmor precession on the current pulse propagation direction relative to the Fe/GaAs crystallographic directions. The results of these experiments are summarized in Fig. 3. The sketch in Fig. 3a indicates the positions of the spatially-separated pump and probe laser spots, which are depicted by large and small circles, respectively. Each color represents a particular pump-probe experiment at the respective position on the disk-shaped device. The effective spin–orbit field ${\varvec{H}}_{{{\varvec{SO}}}}$, which is composed of the Bychkov-Rashba-like (${\varvec{H}}_{{\varvec{R}}}$) and the Dresselhaus-like (${\varvec{H}}_{{\varvec{D}}}$) fields^8^, is schematically shown in Fig. 3b for different lateral photocurrent pulse directions. When the current pulse propagates along the [110] crystallographic direction, which corresponds to the situation when the probe laser spot is spatially displaced relative to the pump spot along this direction, ${\varvec{H}}_{{\varvec{R}}}$ and ${\varvec{H}}_{{\varvec{D}}}$ point along the same direction and, therefore, they sum up. On the contrary, for a current pulse propagating along the $\left[ {\overline{1}10} \right]$ direction, i.e., when the probe laser spot is displaced accordingly, ${\varvec{H}}_{{\varvec{R}}}$ and ${\varvec{H}}_{{\varvec{D}}}$ subtract, which then leads to a correspondingly smaller effective spin–orbit field ${\varvec{H}}_{{{\varvec{SO}}}}$, as indicated in Fig. 3a by a size of the black arrow.

The MO pump-probe traces measured in the places indicated in Fig. 3a are shown in Fig. 3c using the corresponding colors. The curves were fitted by Eq. (1) and the extracted precessional amplitude *A* is shown in Fig. 3d, again using the same color-coding as in panels (a) and (c). Clearly, there is a considerable difference in the precession amplitudes for photocurrent pulses propagating along the [110] and $\left[ {\overline{1}10} \right]$ crystallographic directions. In part, this difference is due to a geometrical factor, since the magnetization, which is aligned along one of the easy axes [as indicated in Fig. 3a], has different angles with ${\varvec{H}}_{{{\varvec{SO}}}}$ generated by current pulses along [110] and $\left[ {\overline{1}10} \right]$ and the corresponding torque is proportional to ${\varvec{M}} \times {\varvec{H}}_{{{\varvec{SO}}}}$. However, taking this geometrical factor into account, the precession amplitude for current pulses propagating along the [110] direction is still about 40% larger than that for pulses propagating along the $\left[ {\overline{1}10} \right]$ direction. This difference cannot be explained by the Oersted magnetic field which is radially symmetric and, therefore, it has an equal amplitude for these two current propagation directions. On the other hand, the difference in the precession amplitudes is in agreement with the difference in the magnitudes of ${\varvec{H}}_{{{\varvec{SO}}}}$ depicted in Fig. 3a, which is an additional independent experimental confirmation that the ultrashort SOT pulses contribute significantly to the excitation of the magnetization precession in our device. The measurements shown in Fig. 3 allowed us to estimate the magnitudes of the Bychkov-Rashba and the Dresselhaus spin–orbit fields by relating them to the magnitude of the Oersted field, which can be calculated from the Biot-Savart law. For a lateral current density of 10^11^ A/m^2^ we obtained $H_{R} \approx 0.2 {\text{mT}}$ and $H_{D} \approx 0.1 {\text{mT}}$. For more details see the Supplementary Note 1.

**Figure 4:**
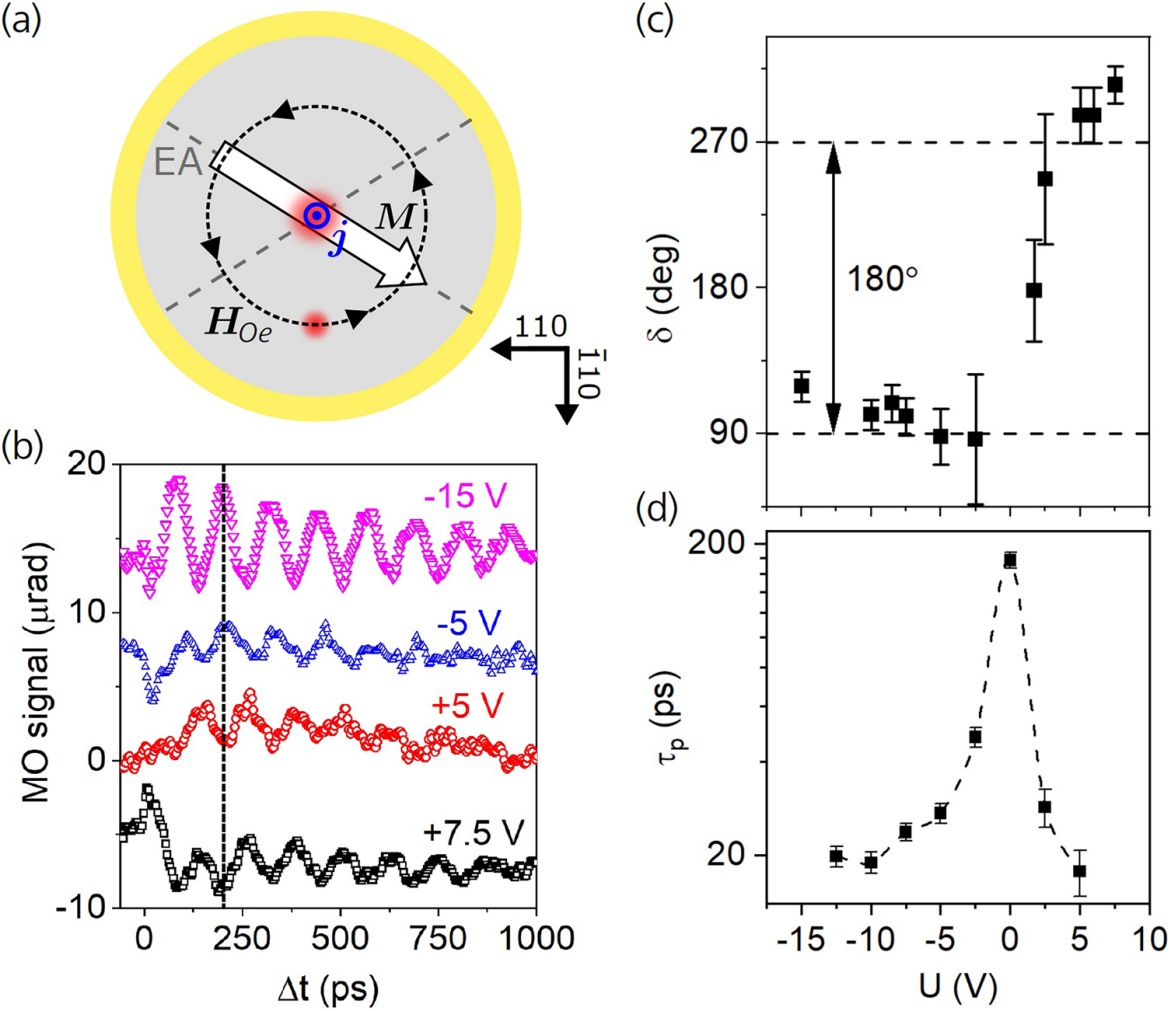
Magnetization precession generated by Oersted field pulses. (**a**) Schematic illustration of the Oersted magnetic field ***H***_*Oe*_ (black circle) induced in the iron film by the vertically propagating current pulse, which is flowing in the pump-illuminated column, with a diameter of ≈ 1 µm, in the photodiode center (larger red circle). Switching the bias polarity reverses the photocurrent and, consequently, the corresponding field ***H***_*Oe*_ acting on magnetization ***M*** within the probe laser spot (smaller red circle), which is displaced by 9 µm away from the pump laser spot along the [$\overline{1}10$] crystallographic direction. **(b)** Magnetization precession induced by ***H***_*Oe*_ measured for several biases without external magnetic field applied; the curves are vertically shifted for clarity. The phase of oscillations depends on the bias polarity, as indicated by the dashed line. **(c)** Bias dependence of the precessional phase *δ* extracted by fitting the data in part (**b**) by Eq. (1) for Δ*t* > 100 ps. **(d)** Bias dependence of the duration of the vertically propagating photocurrent pulse measured by the photocurrent correlation technique. For larger bias magnitudes the photocurrent pulses become considerably shorter than the magnetization precession period, which is the cause of the gradual phase shift of the precession signal observed in (**c**).

To check the consistency of our analysis of the magnetization precession data and, in particular, the interpretation of the initial precession phase, we performed an additional control experiment in a geometry where the SOT magnitude is minimized. As shown in Fig. 3, this is the case when the current pulse propagates along the $\left[ {\overline{1}10} \right]$ direction. In these conditions, the Oersted field torque generated by the vertical current pulse becomes strongest relative to SOT generated by the lateral current pulse and, therefore, a precession phase closer to 90° is expected (see Supplementary Note 1 for details). The experimental geometry is depicted in Fig. 4a. The probe laser spot is displaced along the $\left[ {\overline{1}10} \right]$ direction relative to the current pulse-generating pump laser spot that is placed in the center of the photodiode. The polarity of the photogenerated current pulse and, consequently, of the Oersted field (***H***_*Oe*_) is determined by the polarity of the applied bias voltage. Moreover, the duration of the current pulse can be controlled by the bias magnitude (see Supplementary Note 5), which enabled us to verify experimentally its influence on the initial precession phase.

The pump-probe traces measured for bias voltages ranging from + 7.5 V to − 15 V are shown in Fig. 4b. As indicated by the vertical dashed line, the magnetization precession changes phase by 180° when the bias polarity is reversed. This is expected, since an inversion of the current pulse propagation direction leads to an inversion of ***H***_*Oe*_ and the corresponding torque causes the opposite initial magnetization tilt. The initial precession phase *δ* extracted by fitting the curves with Eq. (1) is shown in Fig. 4c as a function of the applied bias voltage. In contrast to the conditions of Fig. 2, where the dominant contribution to torque was attributed to SOT, a phase of ≈ 90° is now observed for a small reverse bias voltage. This is fully consistent with the ***H***_*Oe*_–induced magnetization precession reported previously for a laser pulse-illuminated Schottky diode^9,20,21^ and also with the phase theoretically predicted for an Oersted field pulse with a duration comparable to or longer than the precession period (see Supplementary Fig. S.1). This is indeed the case for small bias magnitudes in our diode, as is evident from Fig. 4d which shows the current pulse duration measured by the photocurrent correlation technique as a function of the applied bias voltage (see Supplementary Note 5). For larger bias magnitudes the precession phase shifts towards higher values for both bias polarities. This gradual phase shift, which is caused by the decreasing current pulse duration [see Fig. 4d], is in a quantitative agreement with the theoretically calculated phase dependence on pulse duration shown in Supplementary Fig. S.1.

## Discussion

We have demonstrated generation of ultrashort spin–orbit torque pulses at an epitaxial ferromagnetic metal–semiconductor interface by converting femtosecond laser pulses into laterally-oriented high-amplitude current pulses in an electrically biased iron-gallium-arsenide (*p*-*i*-*n*) photodiode. Each ultrafast current pulse, consisting of a sub-picosecond peak and a ~ 10-picosecond tail, excites the precession of iron magnetization directly at the iron-photodiode interface without any dispersion-related pulse broadening. Polarity and lateral flow direction of the current pulses were controlled by the lateral position of a focused laser spot and by the bias voltage on the photodiode. The spin–orbit origin of the torque exciting Larmor precession of the iron magnetization was revealed by a detailed analysis of the precession phase and amplitude at different experimental conditions.

The experimentally observed Larmor precession of the ferromagnetic film with a frequency of ≈ 10 GHz was excited by the ~ 10-picosecond tail of the photogenerated current pulse. However, the much faster sub-picosecond current peak onset of the pulse with up to ~ 100 mA amplitude could be relevant for excitation of antiferromagnetic (AFM) thin films, where the exchange enhanced precession frequencies are in the THz regime^14^. Recent studies have demonstrated manipulation of the AFM Néel vector by passing an electric current through metallic collinear antiferromagnets with locally broken inversion symmetry, such as CuMnAs^22^ or Mn_2_Au^23^. In particular, pulses with current densities of ~ 10^10^–10^11^ A/m^2^ were sufficient to switch the Néel vector of the AFM domains by 180° due to domain wall motions^24,25^. With our device structure, similar or even larger current pulse amplitudes could be generated within sub-ps time, providing a new experimental technique for investigation of ultrafast excitation and switching of magnetic order in metallic AFM thin films^26^.

Moreover, as we demonstrate in Supplementary Note 2, thanks to the optical selection rules in the zinc-blende GaAs heterostructure, our new experimental technique is also able to exploit spin–orbit coupling to convert photon angular momentum to electron spin and, consequently, to generate ultrafast spin-polarized current pulses^16,27,28^. Direct spin transfer from ultrashort optically generated spin current pulse to the AFM layer might be another way of generation of high frequency AFM excitations.

## Methods

### Magneto-optical pump-probe experiment

This technique was used to obtain information about the magnetization (spin) and charge dynamics that is excited in the photodiode directly by laser pulses or by the corresponding photocurrent pulses. Pump-probe experiment is a stroboscopic optical method where the dynamics triggered by a strong (pump) laser pulse is sampled by a time-delayed weaker (probe) laser pulse^29^. We employed a reflection optical geometry and measured the magneto-optical (MO) signal, which corresponds to a probe polarization rotation, and the differential reflectivity d*R*/*R*, which corresponds to a transient change of the probe intensity. Both these signals were measured simultaneously as a function of time delay between the pump and probe pulses using the optical bridge, where they correspond to "difference" and "sum" signals, respectively (see Appendix B in^30^ for details). The measured MO signals did not depend on the polarization of pump pulses; the data depicted in Figs. 2, 3 and 4 were measured for linear polarization. As a light source, we used femtosecond Ti:sapphire laser (Mai Tai, Spectra Physics) producing ≈ 100 fs laser pulses at a repetition rate of 80 MHz. Typically, we employed a geometry with a collinearly propagating pump and probe pulses and a microscopic objective, which was focusing laser beams to a spot size of ≈ 1 μm; see Fig. 1b in^29^. In the experiment, the laser repetition frequency was decreased to 8 MHz by a pulse picker, the laser central wavelength was tuned to 798 nm and spectral filters (NF808-34 in the pump beam and FBH810-10 in the probe beam) were used to generate a quasi-nondegenerate pump and probe pulses with central wavelengths of 794 nm and 804 nm, respectively^29^. We used pump laser power of 230 μW, which corresponds to a fluence of ≈ 1 mJ/cm^2^, and probe laser power was set to 10–20% of the pump power. The data presented in Fig. 2, where a very good spatial overlap of the pump and probe pulses was necessary to suppress the influence of the Oersted field generated by a vertical photocurrent on the detected magnetization dynamics, were measured using an experimental setup with non-collinearly propagating pump and probe pulses where laser beams were focused by a single converging lens to a spot size of ≈ 25 μm; see Fig. 1a in^29^. Here, the laser repetition rate of 80 MHz and the same wavelength of pump and probe pulses of 800 nm were used. The pump power of 60 mW, which corresponds to a fluence of ≈ 0.1 mJ/cm^2^, and probe power of 10% of the pump power were used. The samples were mounted in an optical cryostat and the experiments were performed at a base temperature of 15 K. No external magnetic field was applied during the MO measurements.

### Photocurrent correlation method

For the characterization of ultrashort electrical current pulses generated in our photodiode by femtosecond laser pulses, we used the photocurrent correlation measurement^31^. This technique is sensitive to the nonlinear component of the photodiode response, i.e., to the mutual interaction between two consecutive photo-generated current pulses^31–33^. The nonlinear response of our photodiode to the optical stimulus, which is necessary for this correlation technique to work^33^, is clearly apparent from the VI characteristics shown in Supplementary Fig. S.4b. Similarly like in the case of pump-probe method, we used two mutually time-delayed laser pulses which, however, now have the same intensity. Here, the measured quantity is a time-averaged photocurrent generated in the diode, which was measured by a sensitive ampere-meter as a function of the time delay between the two identical laser pulses. After the impact of the first laser pulse, the photo-generated electron–hole pairs are separated in a strong electric field present in the intrinsic part of the PIN diode and accelerated in the vertical direction towards the opposite electrodes, thus creating a short current pulse [see Fig. 1a]. The presence of the photo-carriers created by the first laser pulse influences both the material properties (e.g., via saturation of absorption) and the diode properties (e.g., by screening of the electric field) until the photo-carriers are removed from the diode by the electric field. The absorption of the second laser pulse is decreased because of the saturation and the electric field, which is weakened by the screening effect, makes the electrical transport slower, which means that more photo-carriers can recombine before reaching the electrodes. Both these effects decrease the total charge which can be transported out of the diode per one pair of laser pulses and, therefore, the average photocurrent measured in the correlation experiment is also decreased. As the influence of the first pulse on the second pulse increases with decreasing time delay between the pulses, the largest decrease of the average photocurrent is expected around the zero time delay.

## Supplementary Information

Supplementary Information.

## Data Availability

The datasets generated and analyzed during the current study are available from the corresponding author on reasonable request.
