# Supplementary material for: Ultrashort spin–orbit torque generated by femtosecond laser pulses
Source: Sci Rep. 2022 Dec 13;12:21550. doi: 10.1038/s41598-022-24808-z (PMC9747954; doi:10.1038/s41598-022-24808-z)
Supplement: Supplementary file 1 — Supplementary Information. [file 41598_2022_24808_MOESM1_ESM.pdf]

Ultrashort spin-orbit torque generated by femtosecond laser pulses

T. Janda,[1,2,a)] T. Ostatnický,[1] P. Němec,[1] E. Schmoranzerová[1], R. Campion,[3] V. Hills,[3] Z. Šobáň,[4] and J. Wunderlich[2,4]

[1]*Faculty of Mathematics and Physics, Charles University, Ke Karlovu 3, 121 16 Prague 2, Czech Republic*

[2]*Institute for Experimental and Applied Physics, University of Regensburg, Universitätsstr. 31, 93053 Regensburg, Germany*

[3]*School of Physics and Astronomy, University of Nottingham, Nottingham, NG7 2RD, UK*

[4]*Institute of Physics ASCR, v.v.i., Cukrovarnická 10, 162 00 Prague 6, Czech Republic*

Supplementary Note 1: Characteristic phase of magnetization precession excited in pump-probe experiment by various mechanisms

The magnetization (*M*) precessional dynamics in ferromagnets is described by the Landau-Lifshitz-Gilbert (LLG) equation that can be expressed in a form [1, 2]:

$$\frac{d\boldsymbol{M}(t)}{dt} = -\mu_0\gamma\left[\boldsymbol{M}(t) \times \boldsymbol{H_{eff}}(t)\right] + \frac{\alpha}{M_S}\left[\boldsymbol{M}(t) \times \frac{d\boldsymbol{M}(t)}{dt}\right], \tag{S1}$$

where γ is the gyromagnetic ratio, α is the Gilbert damping constant, and M_S is saturation magnetization. The effective magnetic field H_{eff} is defined as

$$\boldsymbol{H_{eff}}(t) = \frac{\partial \Phi}{\partial \boldsymbol{M}} , \tag{S2}$$

where Φ is energy density functional that includes Zeeman energy due to the external magnetic field $\boldsymbol{H}_{ext}$ and magnetic anisotropy energy consisting of the magnetocrystalline and shape anisotropy contributions. To describe the later discovered spintronic effects of spin-transfer and spin-orbit torques on the magnetization dynamics, an additional term $\boldsymbol{\tau}$ was added in Eq. (S1) resulting in the Landau-Lifshitz-Gilbert-Slonczewski (LLGS) equation [3]:

$$\frac{d\boldsymbol{M}(t)}{dt} = -\mu_0\gamma\left[\boldsymbol{M}(t) \times \boldsymbol{H_{eff}}(t)\right] + \frac{\alpha}{M_S}\left[\boldsymbol{M}(t) \times \frac{d\boldsymbol{M}(t)}{dt}\right] + \boldsymbol{\tau}(t). \tag{S3}$$

The first term on the right hand side of Eq. (S3) leads to a precession of magnetization about the direction of $\boldsymbol{H}_{eff}$ and the second term leads to relaxation of the magnetization towards this field. Consequently, depending on their symmetry, the spin torques can be sorted as field-like,

of the form $\boldsymbol{\tau}_{FL} \sim \boldsymbol{M} \times \boldsymbol{s}$, and damping-like, of the form $\boldsymbol{\tau}_{DL} \sim \boldsymbol{M} \times (\boldsymbol{M} \times \boldsymbol{s})$ (see Fig. 3 in Ref. 3), with $\boldsymbol{s}$ being the nonequilibrium spin polarization. The damping-like torque is also often called antidamping torque since, depending on the spin polarization direction, it can also turn magnetization away from $\boldsymbol{H}_{eff}$.

After the impact of the pump laser pulse several effects take place, which can modify $\boldsymbol{H}_{eff}$ and induce nonequilibrium spin polarization. Consequently, the corresponding torques $\boldsymbol{M} \times \boldsymbol{H}_{eff}$ and $\boldsymbol{\tau}$ tilt the magnetization away from its equilibrium direction. The direction of this initial tilt influences the phase of the subsequent magnetization precession around the effective field. For example, the field-like and damping-like spin torques are always perpendicular to each other and, consequently, the phases of the magnetization precessions triggered by $\boldsymbol{\tau}_{FL}$ and $\boldsymbol{\tau}_{DL}$ differ by 90° (see Fig. S.1).

The magnetization precession phase is influenced not only by the particular excitation mechanism but also by the duration of the excitation pulse. This effect was discussed in Supplementary Note 1 in Ref. 4 for the extreme cases of very short and very long pulses (compared to the magnetization precession period). For a long pulse, the effective field is modified quasi-permanently and magnetization simply starts precessing around this new equilibrium direction. For a short pulse, the magnetization initially also starts precessing around the same new equilibrium direction. However, this initial stage lasts only for a time window that is much shorter than the magnetization precession period. During this time the magnetization is only slightly tilted and after the effective field is relaxed back to its original direction the magnetization starts its precession in a direction that is perpendicular to its initial tilt, i.e., perpendicular to the precessional motion triggered by the long pulse. Consequently, the magnetization precessions triggered by short and long pulses differ in phase by 90° (see Supplementary Fig. 1 in Ref. 4). The same considerations apply also to other excitation mechanisms that can be described by effective magnetic fields, such as the spin torques (see Supplementary Fig. 2 in Ref. 4).

In the following, we will review various physical phenomena that can be responsible for triggering magnetization precession after absorption of linearly polarized pump laser pulses in our GaAs-PIN/Fe structure and we will discuss how the different effects can be identified in the experimentally measured time-resolved magneto-optical (MO) data. Let us assume that the sample surface lies in the *xy*-plane, magnetization in equilibrium points in the *x*-direction and the pump-laser beam is incident on the sample along the *z*-direction. We also assume, in accord with our experimental conditions, that the experiment is performed in a reflection geometry and that the measured MO signal is due to polar Kerr effect. Consequently, the experimentally measured MO signal is proportional to perpendicular-to-plane component of magnetization M_z. By fitting the measured MO traces by a damped harmonic function in a form of Eq. (1) we can deduce the direction of the initial tilt of magnetization, which is one of the key signatures that can be used to identify the exact mechanism which was responsible for triggering the observed precession of magnetization [4, 5, 6, 7, 8, 9].

(a) Magnetic field pulse

Seemingly, the most straightforward mechanism leading to a precession of magnetization is application of a magnetic field pulse with a sufficiently fast onset. When the pulse rise time is significantly shorter that the magnetization precession period, which is ≈ 100 ps in our Fe thin films, the magnetization can't adiabatically follow the effective field and starts precessing around it. As discussed in detail in Supplementary Notes 6 and 7, the current pulse generated in our Fe/GaAs structure, which consists of photogenerated electrons and holes, has

two stages. Initially the electrons and holes move in vertical direction within the pump-illuminated channel and after reaching the top and bottom electrodes they spread in lateral direction towards the annular contact. The important difference is that during the vertical transport the electrons and holes move in opposite directions, while during the lateral motion they move in the same direction, coupled by the Coulomb interaction. Consequently, the Oersted magnetic fields generated by the electrons and holes during their vertical motion sum up, while the Oersted fields generated during their lateral motion subtract. Thanks to the large aspect ratio of our photodiode with the lateral size being 2 orders of magnitude larger than its thickness, the Oersted fields from the lateral electron and hole currents compensate almost precisely in the iron film and, therefore, they can be neglected.

The precession of magnetization induced by an in-plane Oersted magnetic field pulse, which results from a vertically propagating current pulse generated by a laser pulse excitation, has been previously studied in another vertical photoconductive structure – a Schottky diode [5, 6, 10, 11, 12]. The key fingerprint of this excitation mechanism is a characteristic dependence of the precession amplitude on the mutual spacing of the current pulse generating pump laser spot and the probe laser spot on the sample surface – the precession disappears when pump and probe pulses are overlapped spatially and it is the largest when there is some spacing between them (see Fig. 1(b) in Ref. 10). Furthermore, the resulting precession of magnetization is the largest when the Oersted field is perpendicular to the magnetization orientation and it disappears when these two vectors are parallel (see Fig. 2(a) in Ref. 10). This second feature is, however, true also for other effects, for example, for the current-induced spin-orbit torques discussed in part (c) of this Supplementary Note. Finally, since the Oersted field is oriented in-plane, the magnetization is initially tilted perpendicular to the sample plane, which for longer pulses leads to a precessional phase of 90° or 270°, depending on the mutual position of pump and probe laser spots and the magnetization direction (see Fig. 2 in Ref. 6). For pulses much shorter than the magnetization precession period a phase of 0° or 180° is expected.

(b) Thermal change of magnetic anisotropy

As a matter of fact, the most frequently observed mechanism leading to a pump-induced precession of magnetization is a thermal mechanism, which appears due to different temperature dependencies of the various contributions to the effective field $\boldsymbol{H}_{eff}$. The laser-pulse induced heating leads to a reduction of magnetization and, consequently, to a change in a relative strength of the various terms in $\boldsymbol{H}_{eff}$, which scale differently with M. In particular, the Zeeman term is linear in M, while the magnetic anisotropy energy scales with higher powers of M and, therefore, it is reduced relative to the Zeeman energy after the impact of the pump laser pulse. Consequently, when the external magnetic field $\boldsymbol{H}_{ext}$ is applied at a certain angle with respect to the easy axis (EA) in the studied magnetic material, the laser pulse causes a reorientation of $\boldsymbol{H}_{eff}$, which triggers the magnetization precession (see Fig. 1 in Ref. 13). All MO measurements presented in the main text were performed in zero external magnetic field and, therefore, this particular effect is not relevant in our case. Nevertheless, the thermal mechanism can trigger magnetization precession even in zero magnetic field – in materials, where magnetic anisotropy contains several contributions that have a different temperature dependence. For example, in ferromagnetic semiconductor (Ga,Mn)As the in-plane magnetic anisotropy consists of an uniaxial component, which scales with magnetization as $\sim M^2$, and a biaxial (cubic) component, which scales as $\sim M^4$. Due to the laser-induced heating, the uniaxial anisotropy is enhanced relative to the biaxial anisotropy, which leads to a reorientation of the EAs within the sample plane (see Fig. 3 in Ref. 14). The corresponding torque initially tilts the magnetization out of the sample plane and the subsequent precession around the modified EA continues for hundreds of picoseconds. Heat diffusion from the ferromagnetic film to the

substrate eventually removes the excess heat and the original EA is restored. However, this relaxation usually proceeds at a timescale that is much longer than the magnetization precession period. Consequently, we expect an initial precession phase of 90° (or 270°). We note that our epitaxial Fe film also possesses a biaxial magnetic anisotropy composed from a uniaxial and a cubic component (see Supplementary Note 3 for details) and, therefore, this excitation mechanism might be relevant in our case.

(c) Spin-orbit torque excitation mechanism

Spin-orbit torque (SOT) is generated by a nonequilibrium spin density which occurs due to the spin-orbit interaction. Typically, this phenomenon was studied in nonmagnetic metal (NM)/ferromagnetic metal (FM) bilayers. Here, in-plane charge current induces spin polarization mainly by two mechanisms [15, 16]: the bulk spin Hall effect (SHE) in NM layer and the interfacial inverse spin galvanic effect (iSGE). The SHE converts a charge current into a transverse spin current, inducing a spin accumulation at the NM/FM interface [15]. On the other hand, the iSGE generates a spin density due to spin-orbit coupling directly at the NM/FM interface, which lacks an inversion symmetry [15, 16]. In materials with bulk inversion asymmetry (like GaAs) the spin-orbit coupling has two distinct contributions known as the Rashba and Dresselhaus effects [17], which results in different strengths of SOT for currents flowing along different crystallographic directions [see Fig. 3(b)]. The nonequilibrium spin density $\boldsymbol{s}$ accumulated at the NM/FM interface due to the spin-orbit effects acts as an effective spin-orbit field $\boldsymbol{H}_{SO}$ which exerts a torque on the magnetization in FM. This torque can be decomposed into two parts: the field-like torque $\boldsymbol{\tau}_{FL} \sim \boldsymbol{M} \times \boldsymbol{H}_{SO}$ and the damping-like torque $\boldsymbol{\tau}_{DL} \sim \boldsymbol{M} \times (\boldsymbol{M} \times \boldsymbol{H}_{SO})$. Since the spin-orbit field $\boldsymbol{H}_{SO} \parallel \boldsymbol{s}$ is oriented in-plane, $\boldsymbol{\tau}_{FL}$ causes an initial tilt of the magnetization perpendicular to the sample plane, whereas $\boldsymbol{\tau}_{DL}$ acts within the sample plane [see Fig. 2(a)]. Consequently, for $\boldsymbol{\tau}_{FL}$ we expect the magnetization to precess with an initial phase of 90° (or 270°) and 0° (or 180°) for long and short current pulses, respectively. For $\boldsymbol{\tau}_{DL}$, which is perpendicular to $\boldsymbol{\tau}_{FL}$, the phases will be correspondingly shifted by 90°.

SOT is far less understood than the other mechanisms. It is typically used to electrically manipulate the magnetization in memory devices [15], but it is usually limited to nanosecond timescales. Only very recently, the switching with picosecond-long current pulses due to SOT was demonstrated [18]. Also, an all-optical analog of SOT was reported in ferromagnetic semiconductor GaMnAs [14].

(d) Identification of the excitation mechanisms responsible for the magnetization precession induced by photocurrent pulses in our Fe/GaAs PIN-diode

First of all, in the experimental geometry of Fig. 2, i.e., with the spatially overlapped pump and probe laser pulses, there is no net effect from the Oersted field generated by the vertically propagating current pulse (see Fig. 1(b) in Ref. 10). Furthermore, the absence of the magnetization precession for the case of the laser spots overlapped in the device center [position (ii) in Fig. 2(b)] and the opposite phases of the precessions excited in positions (i) and (iii), as well as the bias dependence of the precessional phase in Fig. 4(c), rule out the thermal change of magnetic anisotropy as a dominant mechanism triggering the precession. Consequently, what remains are only excitation mechanisms that are connected with the generated *lateral* photocurrent pulses. As already mentioned, the effect of the corresponding Oersted field can be neglected due to the compensation of the fields from the electron and hole lateral currents flowing in the top and bottom electrode, respectively. We can also exclude effects due to Joule heating caused by the photocurrent pulses which would be independent of the current direction,

i.e., which would lead to identical precessional phases in positions (i) and (iii) in Fig. 2(c). Therefore, the only remaining excitation mechanism which can appear in the experimental conditions of Fig. 2 and which is compatible with our observations is the spin-orbit torque.

The presence of SOT was further confirmed experimentally by an analysis of the induced magnetization precession. There are two characteristic signatures of SOT that can be identified in the precession phase and amplitude. First, the precession phase of ≈ 135° observed in Fig. 2 can be explained neither by the thermal mechanism nor by the Oersted-field pulse, which would both lead to a precession phase of 90° for current pulses longer than the precession period (see Supplementary Note 5 for the measurements of the current pulse duration). On the other hand, the observed phase can be well explained by SOT, in particular, by the presence of damping-like SOT. As already mentioned, the field-like and damping-like SOTs would induce precessions with phases of 90° and 180°, respectively. Therefore, if both contributions are present, one would observe a phase in the interval of 90° - 180°, depending on the ratio of their amplitudes. This is exactly what we observe in Fig. 2(d), indicating a strong contribution of SOT to the excitation of the magnetization precession. Second, the combination of Rashba and Dresselhaus spin-orbit fields leads to different magnitudes of SOT for currents along different crystallographic directions and, consequently, to differences in the amplitudes of the excited magnetization precessions. This asymmetry is apparent in measurements shown in Fig. 3, where we controlled the direction of the current pulse propagation by positioning the probe laser spot with respect to the pump spot. The observed larger precessional amplitude for current pulses along the [110] direction is in agreement with the larger $\boldsymbol{H_{SO}}$ present in this case, compared to pulses along the $[\bar{1}10]$ direction. Both arguments, based on the precessional phase and amplitude, prove a significant contribution of the ultrashort SOT pulses to the excitation of Larmor precession in our device.

In fact, we can estimate the magnitudes of the Bychkov-Rashba and the Dresselhaus spin-orbit fields based on the measurements shown in Fig. 3. First, the Oersted field magnitude at the distance of the probe laser spot $r = 9$ μm away from the pump laser spot can be calculated using the Biot-Savart law as $B_{Oe} = \mu_0 I_V / 2\pi r$, where I_v is the vertically propagating photocurrent flowing within the pump-illuminated column. Based on the simulation of the current pulse dynamics shown in Fig. 1(d), this corresponds to a field of a few mT during the initial current spike and ~0.1 mT during the current pulse tail, which is more relevant for the Fe precession excitation thanks to its much longer duration and correspondingly larger time-integrated effect on the magnetization. The magnitudes of the Bychkov-Rashba (H_R) and the Dresselhaus (H_D) spin-orbit fields are estimated from our measurements with tightly focused and spatially separated pump and probe laser spots and from the ratio of $H_R/H_D \approx 2$ at epitaxial Fe/GaAs(001) interfaces, as previously determined by spin-orbit ferromagnetic resonance measurements [17]. The measurements with displaced pump and probe laser spots first allowed us to relate the precession amplitude of the iron magnetization to the known Oersted field generated by the vertical photocurrent pulse. By comparing two geometries with orthogonally displaced probe laser spots in which the Bychkov-Rashba and the Dresselhaus fields are aligned parallel and antiparallel, respectively [see Fig. 3(b)], we were able to estimate the approximate magnitudes of the spin-orbit fields as $H_R \approx 0.2$ mT and $H_D \approx 0.1$ mT for a lateral current density of 10^{11} A/m^2. These values are only rough estimates, but they are within the range of spin-orbit fields at epitaxial Fe/GaAs(001) interfaces identified by spin-orbit ferromagnetic resonance measurements [17].

Finally, we will discuss the influence of the current pulse duration on the observed magnetization precession phase. So far, we only considered pulses much shorter or longer than the precession period, for which the initial phase differs by 90°. Obviously, pulse durations in between these two extreme cases lead to some intermediate values of the phase. We performed

a simulation of the magnetization precession excited by a SOT pulse with an instantaneous onset and mono-exponential decay time τ_p. In Fig. S.1 we show the precessional phase δ as a function of τ_p for the case of a sole action of $\boldsymbol{\tau}_{FL}$ and of $\boldsymbol{\tau}_{DL}$. We note that for an action of the Oersted-field-induced torque $\boldsymbol{\tau}_{Oe}$, which is present in the measurements with spatially separated pump and probe laser spots (Figs. 3 and 4), the results are identical as for $\boldsymbol{\tau}_{FL}$. The phases were obtained from the simulated dynamics by fitting the oscillations with a cosine function for times $> 5\tau_p$, when the magnetization is already precessing around the original unperturbed effective field. The simulation confirms the conclusions of parts (a) and (c) regarding the precessional phases that are expected for Oersted-field and SOT pulses much longer / shorter than the magnetization precession period of ≈ 100 ps. In particular, significant phase modification occurs when the pulse duration drops down to ≈ 20 ps, which is in a perfect agreement with our observations of the gradual phase shift for large bias magnitudes in the experiment with ≈ 1 μm-wide spatially-separated laser spots [cf. Figs. 4(c) and 4(d)]. On the other hand, the precessional phase of ≈ 135° observed with the spatially-overlapped ≈ 25 μm-wide laser spots [see Fig. 2(d)] cannot be ascribed to the influence of the current pulse duration, since τ_p in this case is $\gtrsim 300$ ps (see Supplementary Note 5 for details). Consequently, the observed precessional phase can only be explained by an action of SOT, in particular, by a mixture of damping-like and field-like SOTs, as indicated by the experimental data point in Fig. S.1, which represents the phase and pulse duration corresponding to the experimental conditions of Fig. 2.

Supplementary Note 2: Generation of spin-polarized current pulses

The ultrashort current pulses generated in our photodiode can be spin-polarized thanks to the specific band structure of the used semiconductor. Spin-polarized electrons and holes are generated in GaAs by absorption of circularly polarized light due to the effect of optical orientation [19]. The direction of the photoinjected spins is controlled by the direction of light propagation and by its helicity [20]. While the photo-holes lose their spin polarization in less than 100 fs [21], the photo-electrons can maintain their spin polarization for several nanoseconds in optimally n-doped semiconductors [22, 23] and, therefore, can exert a torque on magnetization. This was experimentally demonstrated in a ferromagnetic semiconductor GaMnAs [24], where the spin-polarized electrons were generated directly in the magnetic material.

Our device design, where a vertical photodiode structure made of GaAs semiconductor is combined with a thin metallic ferromagnetic layer, offers a possibility to inject ultrashort spin-polarized current pulses also to metals where spins can't be generated directly by light absorption. Under a reverse bias the photo-electrons generated in the intrinsic layer of the diode are accelerated towards the magnetic layer, which is deposited directly on top of the diode, creating an ultrashort spin-polarized current pulse with a sub-picosecond onset. We note that this method of generation of ultrashort spin-polarized electrical pulses using circularly polarized laser pulses can be applied neither in a standard lateral photoconductive switch (Auston switch) [25, 26] nor in a Schottky diode [10], which have been previously used to optically generate picosecond current pulses. In the Auston switch the active area is formed by a gap in a metal electrode, which is deposited on top of a semi-insulating semiconductor substrate, where electric bias is applied. Illumination of the gap by a femtosecond laser pulse (with an appropriate wavelength) generates electron-hole pairs in the substrate which enable temporarily a current to flow across the gap. In the case of circularly polarized laser pulses the corresponding current pulses can be spin-polarized. The photogenerated current pulses are then delivered to the investigated magnetic layer via an attached coplanar waveguide, typically over a distance of several tens of micrometers [18, 27, 28, 29]. However, the spin polarization of the

photo-electrons is lost during the transport in the metal waveguide already after tens of nanometers [4, 30]. This spatial separation of the injection point of spin-polarized carriers and the position of investigated magnetic layer is not an issue in the Schottky diode, where the metal layer is deposited directly on top of the semiconductor. Nevertheless, in the typically used Schottky barrier formed between the n-type semiconductor and the metal, the built-in electric field accelerates the spin-polarized electrons away from the (magnetic) metal (see Fig. 3 in Ref. 10). Here, the charge current pulse is transported by holes, which are not spin-polarized due to their ultrafast (sub-100 fs) spin relaxation time [21].

The long-lived spin polarization of electrons photo-generated in our device was confirmed by time-resolved MO measurements performed using the pump-probe setup (see Methods section in the Main text). In Fig. S.2(a) we show the MO signals measured for opposite helicities of the pump laser pulses both with and without external magnetic field $\boldsymbol{H}_{ext}$. For $\boldsymbol{H}_{ext}$ applied in the sample plane, i.e., perpendicular to the photoinjected spin, the spins are forced to precess around $\boldsymbol{H}_{ext}$. Consequently, the measured MO signal, which is proportional to the spin polarization projection to the probe beam propagation direction, gives rise to the oscillatory signals. For opposite laser helicities the curves are phase-shifted by 180° due to the opposite directions of the spin at the instant of photo-generation. For $H_{ext} = 0$ the electron spin only decays without precession, which manifests as the envelopes of the oscillating curves in Fig. S.2(a). From the measured linear dependence of the oscillatory (Larmor) frequency on H_{ext} [see Fig. S.2(b)] we inferred the magnitude of the g-factor of 0.39 ± 0.02, which confirms that the spin carriers are electrons in GaAs [31]. The measured damping of the MO signal can be fitted by a double-exponential decay where the shorter and the longer time constants can be attributed to the lifetime of photoinjected carriers and to the electron transverse spin coherence time T_2^*, respectively (see Eq. (1) in Ref. 23 and the adjacent discussion). From the context of this paper, the most important observation is that the measured electron spin coherence time $T_2^* \sim 1.5$ ns is considerably longer than the duration of electrical pulses (see Fig. S.5), i.e., that the photoinjected electrons are spin polarized in GaAs when they contribute to the electrical pulse.

In the experiment reported in the Main paper, the iron magnetization precession did not depend on the polarization of pump laser pulses. The absence of pump helicity-dependent oscillatory MO signal indicates that the spin polarization of the current pulses was not sufficient to overcome the large demagnetizing field of the iron film, which acts against the spin transfer torque-induced perpendicular-to-plane tilt of the magnetization, which is triggering the precession [8, 24, 32, 33]. Alternatively, the spin polarization of the current pulses might have been reduced while crossing the Schottky barrier at the n-GaAs/Fe interface [34] making the efficiency of the spin transfer torque too low to be experimentally observable.

Supplementary Note 3: Magnetic anisotropy of Fe/GaAs epilayer

The studied sample contains iron film with a thickness of 2 nm, which corresponds to approx. 14 monolayers (ML) of Fe, that is grown on GaAs(001). Magnetization in the sample lies in the film plane due to a strong (≈ 2 T) demagnetizing field. The in-plane magnetic anisotropy of the film was characterized by a SQUID magnetometer. The $M(H_{ext})$ dependencies measured along three different crystallographic directions are shown in Fig. S.3(a). By fitting the data to the theoretical expressions given in Ref. 35 we identified an uniaxial anisotropy component with easy axis (EA) along the [110] direction and anisotropy field of $\mu_0 H_u = -13 \pm 3$ mT, and a cubic component with EAs along [100] and [010] directions and anisotropy field of $\mu_0 H_c = 30 \pm 6$ mT. This magnetic anisotropy is quite unusual for such an ultrathin film

because in very thin films ($\lesssim$ 25 ML) the uniaxial anisotropy typically dominates [36]. A possible explanation is the influence of interfaces. It is known that the cubic anisotropy has both surface and volume contributions while the uniaxial one originates purely from the interfaces (both Fe/GaAs and Fe/cap). These surface anisotropies are very sensitive to a surface quality, termination of the substrate, material of the capping layer or eventual mixing of Fe with the materials at the interfaces. These surface effects become increasingly important with decreasing the thickness of the Fe layer and can significantly modify the overall magnetic anisotropy in very thin films. Presumably, they are responsible for the observed magnetic anisotropy in our sample.

To verify independently the deduced magnetic anisotropy, we measured in magneto-optical pump-probe experiment a dependence of magnetization precession frequency on external magnetic field applied along the $[\bar{1}10]$ direction. For fields larger than ≈ 150 mT, the magnetization points along the field direction and the precessional frequency follows the Kittel formula [37]

$$f = \frac{\mu_0 \gamma}{2\pi} \sqrt{\left(H_{ext} + M_S^{eff} + H_C + 2H_U\right)\left(H_{ext} - 2H_C + 2H_U\right)}, \qquad \text{(S4)}$$

where $\gamma = (g\mu_B)/\hbar$ is the gyromagnetic ratio, g is the Landé g-factor, μ_B is the Bohr magneton, and $\hbar$ is the reduced Planck constant. H_{ext} is the magnitude of the external magnetic field, H_c and H_u are the anisotropy fields corresponding to the in-plane cubic and uniaxial contributions, respectively. The effective magnetization $M_S^{eff} = M_S - 2H_{out}$ contains the contributions from the shape anisotropy (demagnetizing field), which is connected with the saturation magnetization M_s, and the out-of-plane uniaxial anisotropy H_{out}. While in the first bracket in Eq. (S4) the material anisotropy fields do not play an important role (as it is dominated by the demagnetizing field $\mu_0 M_s \approx 2$ T), in the second bracket in Eq. (S4) the magnitude of H_c and H_u have a significant influence. Therefore, we define an effective anisotropy field $H_a^{eff} = H_c - H_u$ and the fitting formula reduces to

$$f = \frac{\mu_0 \gamma}{2\pi} \sqrt{\left(H_{ext} + M_S^{eff} + H_a^{eff} + 3H_U\right)\left(H_{ext} - 2H_a^{eff}\right)}. \qquad \text{(S5)}$$

Because the fitting procedure is almost insensitive to the precise value of H_u in the first bracket in Eq. (S5), we set there $\mu_0 H_u = -13$ mT, as measured by SQUID. The measured dependence of precession frequency on H_{ext} is shown in Fig. S.3(b) together with the corresponding fit. The obtained g-factor of 2.1 exactly agrees with the value reported in literature for Fe [35], confirming that the oscillatory MO signal corresponds to the precession of magnetization in iron. The effective anisotropy field $\mu_0 H_a^{eff} = 36 \pm 4$ mT also agrees very well with the value obtained by SQUID ($\mu_0 H_c$ - $\mu_0 H_u$ = 43 ± 9 mT). Finally, the obtained effective magnetization $\mu_0 M_S^{eff} = 1.67 \pm 0.25$ T gives for a typical value of $\mu_0 H_{out}$ = 235 mT [36] a value $\mu_0 M_s$ = 2.14 T, which perfectly agrees with the saturation magnetization of bulk Fe [36].

Supplementary Note 4: Electrical properties of Fe/GaAs photodiode

The V-I characteristic of our photodiode measured in darkness is shown in Fig. S.4(a). The diode can withstand large bias magnitudes of both polarities, unlike previously used Schottky diodes [10, 11]. The ability to reverse the direction of electric field in the intrinsic layer of our diode and, consequently, the direction of the photocurrent pulse propagation was important to identify the effects of the current-induced torques on iron magnetization.

The behavior of the photodiode under illumination is shown in Fig. S.4(b). The individual V-I curves were measured using a single fs-laser beam focused to the device center with the laser fluency increasing in constant steps of 0.45 mJ/cm2. Apparently, the measured photocurrent increases sub-linearly, which indicates a decreasing charge collection efficiency with an increasing photo-carrier density. This is caused mainly by the interplay between the internal electric field and the photo-generated charge [38, 39], as explained in detail in Supplementary Note 6. This non-linear behavior is necessary for the photocurrent correlation technique to work.

Supplementary Note 5: Characterization of the electrical pulses by photocurrent correlation technique

As mentioned in the Main text, the later stages of the current pulse decay in the presence of the screening electric field were investigated by the photocurrent correlation technique (see the Methods section). In Fig. S.5(a) we show the correlation curves measured with the laser spots having a diameter of ≈ 1 µm, which corresponds to the experimental conditions of Figs. 3 and 4 in the Main text. The measured photocurrent is plotted relative to its value at zero time delay for different external biases of both forward and reverse polarities. As expected, the photocurrent is reduced around $\Delta t = 0$ (see Methods for details). Note that in our convention, the current is negative for reverse (negative) bias and positive for forward (positive) bias, i.e., in both cases the photocurrent magnitude decreases for small time delays.

The interpretation of the measured correlation signal is not straightforward. The theoretical analyses performed in Refs. 39 and 40 link the temporal profile of a single photocurrent pulse to the profile of the measured time-averaged correlation signal. In their case of a lateral photoconductive switch, the double-exponential profile of the correlation signal was attributed to the exponential decay of the photocarriers (and the corresponding photocurrent) via trapping and recombination. As shown in Fig. S.5(a), the photocurrent correlation data from our PIN diode can be fitted well by a single-exponential model

$$I_{ph}(\Delta t) = I_0 + I_p\, e^{-\frac{\Delta t}{\tau_p}} \qquad \text{(S6)}$$

for larger reverse biases. Here, I_0 is an offset that corresponds to a dark current plus the photocurrent for well time-separated laser pulses, I_p is the photocurrent drop induced by overlapping the two current pulses in time, i.e., it is a measure of the nonlinearity of the system, and τ_p corresponds to the decay time of the photocurrent pulse. For a low or zero bias, the electric field screening is more pronounced, which might explain the slightly distorted correlation curve. However, based on the aforementioned theoretical analyses, we assume that also in our case the decay time of the photocurrent correlation reflects the overall duration of the underlying ultrashort photocurrent pulses. This was additionally supported by the performed numerical simulations (see Supplementary Note 6 for details). In the case of a forward bias, the

photocurrent correlation obviously contains an additional component with an opposite polarity, i.e., a component which corresponds to an effect increasing the photocurrent with decreasing the time delay. We ascribe this contribution to the effect of heating. Each laser pulse absorbed in the diode heats the semiconductor, which temporarily increases its conductivity and, therefore, the forward current supplied by the external electrical source (we apply a constant voltage). Since the conductivity of an intrinsic semiconductor increases super-linearly with temperature, $\sigma \sim \exp(-E_g/(2k_BT))$, the closer the two laser pulses are in time, the larger is the temperature increase and correspondingly larger is the average current flowing through the diode. We took into account the heating effect by adding a second exponential term, $\exp(-|\Delta t|/\tau_h)$, to Eq. (S6), where τ_h corresponds to the time-scale at which the pulse-induced heat is dissipated from the illuminated spot. The decay times τ_p determined by the fitting procedure are shown in Fig. 4(d) in the Main text as a function of the applied bias. Clearly, the decay time decreases rapidly with increasing bias and saturates at ≈ 25 ps for biases with magnitude larger than 5 V. This is expected, as the stronger electric field depletes the photo-generated charge faster. From the fitting we obtained also the heating-related time constant $\tau_h = 210 \pm 30$ ps. We note that the deduced decay time is limited mainly by a transport of the screening charge from the central area of the device to the circular draining electrode (see Supplementary Note 6 for a detailed discussion). Therefore, shorter and stronger current pulses can be expected in devices of smaller diameter.

In Fig. S.5(b) we show the photocurrent correlation curves obtained with laser spots having a diameter of ≈ 25 μm, which corresponds to the experimental conditions of Fig. 2 in the Main text. Apparently, the correlation signal decays much slower than in the case of the smaller laser spots. We fitted the correlation curve obtained for a bias of -10 V by Eq. (S6) supplemented by an additional linear function to account for the linear slope. This linear background was probably caused by the fact that the two time-delayed laser pulses were not absolutely identical or by slight changes in the properties of one of the laser spots when the time delay was changed by the optical delay line, which only become noticeable for a large range of time delays. The exponential decay time obtained by fitting of ≈ 350 ± 70 ps is an order of magnitude longer than that for current pulses generated by the small laser spots. It is also significantly longer than the magnetization precession time and, therefore, the measurements with the overlapped pump and probe laser spots shown in Fig. 2 were performed in the limit of a "long" current pulse, in the context of the discussion in Supplementary Note 1. The longer duration of the current pulses generated by the 25 μm-wide laser spot is caused by much larger amount of photogenerated charge and, consequently, more pronounced screening effects that prevent a fast drainage of the photocarriers from the device. The large amount of photogenerated charge is also apparent from the measured time-averaged photocurrent magnitudes of several mA compared to tens of μA in the case of 1 μm-wide laser spots.

Supplementary Note 6: Theoretical modelling of photocurrent pulses

The system under consideration is in the initial state composed of electron-hole pairs at low temperature. The calculated density of photo-excited pairs is below the exciton Mott density and the thermal energy is below the exciton binding energy. The electric field which is a sum of the external and the built-in fields is, however, large enough to prevent electron-hole binding so the system may be regarded fully as a two-component classical plasma without quantum correlations caused by the overlap of the wavefunctions between individual electrons and holes. The only microscopic effect, reflected in the systems dynamics, is then the electron-hole recombination.

We calculated the initial distribution of the electron-hole density, considering the propagation of a laser pulse at a given wavelength within a saturable absorbing medium. The saturation density of electron-hole pairs was set to 3×10^{17} cm^{-3}, according to a microscopic estimate based on a three-band model of *i*-GaAs. The subsequent dynamics of the two-component plasma was simulated with a FDTD algorithm using a two-dimensional mesh. Thanks to the rotational symmetry, all variables are constant over the angular coordinate in the cylindrical coordinate system and, therefore, they depend only upon the longitudinal and radial positions, which are denoted hereafter as vertical and lateral, respectively – see Fig. 1(a). The *p*- and *n*-doped layers were considered as two single layers between whom the *i*-GaAs area was sandwiched. The topmost ultrathin (2 nm) Fe layer was not included as a separate layer in the simulations because its effect on the overall systems dynamics is not considerable. The reason is that the magnitude of the lateral current is in our particular geometry determined solely by the small conductivity of the bottom *p*-doped layer due to the *long-range* attractive Coulomb force acting over the micrometer thick intrinsic GaAs layer between the laterally propagating electron and hole currents. An effective increase of the conductivity of the topmost layer has, therefore, no effect at all.

We used the following parameters in the simulations: the device radius of 50 μm was divided to 256 cells, the i-GaAs layer thickness of 1 μm was divided to 24 cells with the electron and hole mobilities 20 000 cm^2/Vs and 1 000 cm^2/Vs, respectively. For *p*-GaAs the layer thickness, majority charge concentration and its mobility were 500 nm, 2×10^{18} cm^{-3} and 200 cm^2/Vs, respectively. Finally, the parameters for the *n*-GaAs layer were 20 nm, 5×10^{18} cm^{-3} and 2 000 cm^2/Vs [41, 42]. The electron-hole recombination time considered in our simulations was 100 ps. The device with two electrodes under the reverse bias may be regarded as a charged capacitor with an additional built-in field due to the *p-i-n* junction. The photo-injected charges generated between the electrodes spatially separate to a positively and a negatively charged cloud, each of them traveling towards the oppositely charged electrode (i.e., electrons in the direction of the *n*-GaAs for the reverse bias). This motion results in a screening of the internal field and in slowing down the motion of the screened charges. The charge which eventually hits the electrodes discharges the capacitor and is quickly drained to the electric circuit due to the external voltage, thus rebuilding the internal field and transferring the rest of the charge towards the electrodes. In our device, however, the draining of the charges from electrodes is not immediate as they need to travel from the illuminated center of the disc to its edge to reach the draining contact. The trajectory of each electron (hole) can be, therefore, separated to the part where it propagates vertically towards one of the electrodes and a subsequent lateral motion to the edge of the structure – see Fig. 1(a). This separation of current to two distinct routes, which is one of the major assets in our device structure, is justified by the proportions of the system: ratio of the radius to the height is 50:1 and also the width of the excitation spot is much smaller than the device radius. The vertical electric field, even when screened by the charges in the electrodes, therefore represents the major driving force and the horizontal electrical field becomes important only when the vertical one vanishes.

Results of the numerical calculations are shown in Fig. S.6. The distinct stages of the systems evolution can be well recognized in Fig. S.6(a) where we plot the evolution of the vertical electron current. In the initial phase, there is a large current immediately after the photo-generation of charge carriers which decreases non-exponentially due to a fast buildup of a screening in the electrodes. Then, after several picoseconds, the screening charge slowly leaves the photoexcited central area of the device while it is being instantly refilled by the vertical current. Finally, after all the charge is drained from and/or recombines in the *i*-GaAs area, the vertical current ceases. In Fig. S.6(b) the magnitude of the lateral current at the edge of the photoexcited area reveals exactly the same behavior since once the charges enter the electrode,

they migrate towards the edge of the device. The only difference is that there is a residual current due to the screening charge after the cease of the vertical current. The lateral current pulse spreads in time while propagating towards the drain contact – this feature is depicted in Fig. S.6(c) where we observe the initial fast (≈ 50 ps) current peak only at positions not further than 25 μm from the device center. Closer to the device edge, the current pulse is characterized by a slow initial build-up and then a monoexponential decay. The simulation of the photocurrent correlation measurement is shown in Fig. S.6(d). As already described, its initial fade-out is an effect of the removal of the electric charge from the optically active region, which is driven by the recombination plus its transport to the electrodes and further to the drain contacts. The time constant of the exponential fit of the correlation functions, which is shown in Fig. 4(d) as a characterization parameter describing the experimentally measured photocurrent correlation curves, gives an estimate of the overall length of the vertical current pulse [cf. Figs. S.6(a) and S.6(d)]. Note, however, that – unlike in transient reflectivity measurements [see Fig. 1(c)] – the initial ultrafast stage of the vertical current [see the Inset in Fig. S.6(a)] is not apparent in the correlation function due to rather different dynamics of electrons and holes. Further theoretical analysis of the properties of generated electrical pulses and resulting magnetic and SOT pulses, including their optimizations by changing the device structure and dimensions and/or properties of the excitation laser pulses, are provided in Supplementary Note 7.

Supplementary Note 7: Theoretical predictions about possible future device optimizations

In the current dynamics of the studied device, there are several time constants of interest. Here we discuss them separately with a focus on eventual future experiment optimizations by changing the device structure and dimensions and/or properties of the excitation laser pulses. In the discussion we consider only the consequences for the Oersted-field pulses generated by the vertical current pulses. However, very similar conclusions apply also to the SOT pulses induced by the lateral current pulses, as both the Oersted field and $\boldsymbol{H}_{SO}$ are proportional to the current density and the temporal profile of the lateral current pulse at the edge of the illuminated area is very similar to that of the vertical current pulse [cf. Figs. S.6(a) and S.6(b)].

(a) Initial fast decay of vertical current due to electric field screening

As discussed in Supplementary Note 6, the electron-hole cloud is created within the electric potential between charged electrodes of the p-i-n structure and the charge separation begins immediately after the photo-excitation, giving rise to the vertical current. To reveal the intrinsic speed limits in this device structure, we consider a delta function-like temporal profile of the excitation pulse for the purpose of our theoretical analysis. The expressions below are derived, based upon the consideration of the expression for the drift current density $j = -en\mu E$ where e is the (positive) electron charge, n is the photo-excited electron density, μ is the electron mobility in the i-GaAs layer and E is the (built-in plus external) electric field intensity. This assumption is not always fulfilled due to ultrafast dynamics of our system whose characteristic time constant is shorter than the electron scattering time. Therefore, the results calculated by this simplified approach will be used solely as an estimation of the order of magnitude of the variables of interest. The vertical current results in a local charge accumulation in the electrodes and a subsequent screening of the electric field which slows down the motion of charges. Taking into account that the initial electron motion occurs in a dense electron-hole plasma, where the electron scattering time decreases approximatively by a factor of 5 [43], we can define a reduced mobility $\bar{\mu}$ ($\bar{\mu} \approx \mu/5 \sim 4000$ cm^2/Vs) thus resulting in $j = -e\bar{\mu}nE$. The temporal change of the surface electric charge density σ follows the continuity equation and the temporal

change of the electric field in the close vicinity of the electrode is then $\dot{E} = \dot{\sigma}/\varepsilon = j/\varepsilon = -e\bar{\mu}nE/\varepsilon$, where ε is the material electric permittivity. Finally, we can make an estimate of the vertical current relaxation time in the form:

$$T_{\mathrm{vert}} = \frac{\varepsilon}{e\bar{\mu}n}, \qquad \text{(S7)}$$

assuming n = const. This is not, however, fully true since we did not take into account the full spatio-temporal evolution of both the electron and hole density – the electron density in Eq. (S7) is over-estimated and, therefore, the time constant represents the lower limit. Putting the parameters considered in numerical calculations presented in Supplementary Note 6 to Eq. (S7), we arrive at the estimate of the order of magnitude $T_{\mathrm{vert}} \sim 10$ fs. As this value is smaller than the electron scattering time of 150 fs [43], we conclude that the vertical current dynamics is more complex than a mono-exponential decay assumed above. Nevertheless, the deduced time constant represents still a good estimate of the initial decay order of magnitude for the vertical current pulse. For ultrafast applications, interesting is not only the current but also the total charge transferred during this fast initial current phase. This can be easily estimated based on the charge surface density required to screen the field. For the external bias $U_0 = -10$ V, device height h = 1 μm and radius of excited area R_{exc} = 2.2 μm, the number of electrons is $\pi R_{\mathrm{exc}}^2 \varepsilon\, |U_0|/(eh) \approx 10^5$, which corresponds to charge of $\approx 10^{-14}$ C and current amplitude of up to ≈ 1 A for the delta function-like laser pulse or ≈ 100 mA for the 100 fs long laser pulse used in the experiment. Clearly, the very fast initial current impulse has a drawback in a rather small amount of transferred charge and subsequent slow dynamics where the majority of electrons reach the electrode. Note that we analyze only the electrons here: holes have much smaller mobility and, therefore, the initial phase of current is covered solely by the transport of electrons.

It follows from this analysis that the amount of the charge which is transferred within this very initial phase can be controlled by the external voltage and the laser spot size. From the point of view of possible applications, the question is whether we can modify the experimental conditions in order to achieve an ultrafast Oersted magnetic field of a desired peak value and time-integrated magnitude. We can estimate the temporal profile of the initial current in the form:

$$I(t) = \frac{\pi R_{\mathrm{exc}}^2 e\bar{\mu}n|U_0|}{h} \exp[-t/T_{\mathrm{vert}}]\,. \qquad \text{(S8)}$$

We remind here that the above formula is an estimate of the very initial current peak with ultrafast (subpicosecond) relaxation time that is followed by the further slower system evolution [characterized by a flat part starting at ~ 10 ps in Fig. S.6(a)], which will be discussed below and which is not covered by Eq. (S8). It is, nevertheless, possible to optically excite such low particle density that there are no electrons left in the initially excited area after the complete screening buildup. The vertical current would immediately drop down to zero after this ultrafast initial phase which is a scenario perfectly suitable for the ultrafast manipulation of magnetic moments. For example, the Oersted field would then last less than one picosecond only. The external bias U_0 is exactly screened out after collection of the photo-created electron density n_{max} into the electrode, where

$$n_{\max} = \frac{\varepsilon|U_0|}{eh^2}. \tag{S9}$$

Inserting this into Eq. (S8) we can express the magnetic field intensity H in the distance r from a current filament as $H=I/2\pi r$. The maximum field amplitude is achieved in a close vicinity of the excited spot, i.e., for $r = R_{\mathrm{exc}}$. Considering the infinitely short delta function-like excitation pulse and performing some algebra, we get the estimates for the peak and integrated magnetic field intensity as:

$$H_{\mathrm{peak}} = \frac{R_{\mathrm{exc}}\bar{\mu}\varepsilon U_0^2}{2h^3}\ , \tag{S10}$$

$$H_{\mathrm{integrated}} = \int_0^\infty H(t)dt = \frac{R_{\mathrm{exc}}|U_0|\varepsilon}{2h}\ . \tag{S11}$$

In a real experiment, however, the time relaxation constant introduced in Eq. (S7) is much shorter than the actual laser pulse duration and the vertical current pulse duration is therefore determined by the temporal profile of the laser pulse. Taking into account parameters of our simulations, external bias $U_0 = -10$ V and optical pulse length 0.1 ps, we estimate (by considering the convolution) that the peak Oersted field is 20 Oe. As Eq. (S10) predicts the peak field of 60 Oe, we may conclude that Eq. (S10) gives a good estimate of the field upper limit for the subpicosecond optical excitation.

The above expressions may be interpreted in a way that we can independently tune the peak Oersted field and its action by properly setting the radius of the excited area and by setting the external bias. Further possibility is to control the peak field by a proper tuning of the excitation density. If excitation density lower than n_{max}, which is given by Eq. (S9), is used, a lower peak Oersted field is obtained without a change of the ultrafast field temporal profile. On the other hand, if higher excitation density is used, the peak field would be enhanced as compared to Eq. (S10) but, simultaneously, the magnetic pulse would be longer due to a slow discharging of the structure. Moreover, if very short magnetic pulses are required for a certain application, it is essential to use excitation femtosecond laser pulses as short as possible because their duration would have the dominant effect for the resulting magnetic pulse duration.

(b) Discharging phase of vertical current

The slow discharging of the photo-excited *i*-GaAs layer has a complex non-exponential dynamics determined by a spatial distribution of the electric field and charges. The current magnitude is proportional to the external bias but not to the photo-created charge density. Therefore, the temporal width of the vertical current should be proportional to the total amount of excited charge carriers and inversely proportional to the applied bias, as confirmed by our numerical calculations.

As already mentioned above, we can avoid this intermediate slow part of the dynamics by a photo-creation of only a limited density of charged particles, which is described by Eq. (S9). On the other hand, for certain applications, the sub-picosecond duration of the Oersted field pulse might not be necessary and achieving a strong field pulse, with a time integral larger than the value given by Eq. (S11) for a maximum allowed external bias U_0, might be advantageous. In such a case, the electron density exceeding n_{max} can be used. Consequently, it

results in a slow decay of both the electron density in the i-GaAs layer and the vertical current for times exceeding ~ 10 ps, as depicted in Fig. S.7(b) and Fig. S.6(a), respectively. Here, the temporal length of the Oersted field pulse reveals a nontrivial dependency on the external bias. The time-integral of the magnetic field intensity can be expressed from the time integral of the vertical current: as this is the total electron charge in the structure, we may write directly (assuming a negligible electron-hole recombination):

$$H_{\text{integrated}} = \frac{R_{\text{exc}} enh}{2} \,. \tag{S12}$$

For the photo-created charge density 3×10^{17} cm^{-3} used in our simulations and the external bias $U_0 = -10$ V, the overall time-integrated field is equivalent to the mean field intensity 3.7 Oe acting for 60 ps.

(c) Removal of screening charge by lateral current

The dynamics at longer time delays, when all the charges are drained out of the photo-active region, is driven by the electric field inside the electrodes acting in the radial direction of the device. Keeping in mind that the width of the device is much larger than its height, we derived an approximate expression for the time constant for the residual charge transport from the device:

$$T_{\text{res}} = \frac{\varepsilon R_0^2}{4eh}\left[\frac{1}{n_1 t_1 \mu_1} + \frac{1}{n_2 t_2 \mu_2}\right], \tag{S13}$$

where R_0 is the device radius, and the parameters of the respective doped layers (j = 1,2) are the acceptor/donor concentration n_j, the layer thickness t_j and the majority carrier mobility μ_j. For a multilayered electrode, we sum up contributions to the overall conductivity from all participating layers in the particular denominator in Eq. (S13). This expression illustrates the major contribution of the slower holes to the overall charge dynamics. Consequently, increasing the conductivity of the electron channel does not speed up the overall charge transport. Moreover, this time constant is independent of the external bias, as confirmed by our numerical calculations [see the final stages of the photocurrent correlation curves shown in Fig. S.7(a)].

The time constant T_{res} affects also the current dynamics in the intermediate phase because it determines the rate at which the screening charge is removed. Therefore, fabrication of devices with a smaller diameter and/or higher hole concentration in the p-doped layer would lead to the current speedup. Smaller diameter of devices would also help to deliver shorter pulses to the regions close to ring drain contacts, as depicted in Fig. S.6(c). The other promising way to speed-up the current in the device is to increase the i-GaAs layer thickness. The time constant T_{res} would decrease and the total charge stored in the device, and eventually released to the electric circuit, would increase due to the increased volume of the photo-active region.

(d) Photocurrent correlation measurements

The principle of a photocurrent correlation measurement is based upon the influence of the excess charge, which is present in the device due to absorption of the first laser pulse, on

the properties and propagation of the photocurrent pulse induced by the second laser pulse. First of all, the charge occupying the *i*-GaAs absorbing layer inhibits an additional excitation of electron-hole pairs and, therefore, it reduces the total charge which is injected in an electric circuit. In addition, charges present in the electrodes after their drainage from the optically active *i*-GaAs region influence the correlation curves since they reduce the electric field in the device, slow down the spatial separation of the carriers injected by the second pulse and, finally, they cause an excessive recombination as compared to the situation without any preceding laser pulse. In principle, the correlation curves should reflect all changes in the charge distribution in the device throughout the whole time interval between the photo-creation of charges till its full discharge to an external electric circuit. Therefore, all three distinct regimes of the charge dynamics, which were described above, should be visible in the photocurrent correlation curves. These particular regimes have, however, rather different imprints in the curves and, therefore, they will not be apparent with the same magnitude.

The fast initial buildup of the screening charge is connected with the drainage of carriers from the *i*-GaAs layer. Considering delta function-like optical excitation pulse (i.e., an instantaneous creation of the electron and hole populations), a significant fraction of electrons leaves the *i*-GaAs part of the sample on sub-picosecond time scales [see inset in Fig. S.6(a)]. On the contrary, the holes have much lower mobility and, therefore, they cannot quit the photo-excited area at the time scales given by Eq. (S7). As a result, optical absorption remains saturated even at the end of this initial ultrafast part of the current dynamics and the initial current dynamics is not apparent in the photocurrent correlation curves, as seen both in the experimental [Fig. S.5] and theoretical [Fig. S.6(d)] data. The second part of the dynamics is connected with a simultaneous drainage of electrons and holes from the optically active *i*-GaAs layer in the device structure. Each drained electron-hole pair allows absorption of an additional photon from the second laser pulse. Due to this, this process is visible as an increase of the total photocurrent in the photocurrent correlation curves. In fact, this part of the dynamics, when the *i*-GaAs is being discharged [see Fig. S.7(b)], covers the main part of the correlation curve [see Fig. S.7(a)]. In the last stage of the current dynamics, when charges have been drained from *i*-GaAs and when they migrate in the radial direction, the photocurrent correlation curves display a joint effect of the screening by the residual charges in the electrodes and of electron-hole recombination. As the amount of the screening charge is only a few percent of the total photo-excited charge in our simulations, the last part of the correlation has only a very little relative magnitude [see Fig. S.7(a)] and could be hidden by a noise in the experimental data.

REFERENCES

[1] J. Miltat, G. Albuquerque, and A. Thiaville, *An introduction to micromagnetics in the dynamic regime*, in Spin dynamics in confined magnetic structures I, edited by B. Hillebrands and K. Ounadjela, Springer, Berlin, 2002, vol. 83 of Topics in applied physics.

[2] C.S. Davies, K.H. Prabhakara, M.D. Davydova, K.A. Zvezdin, T. B. Shapaeva, S. Wang, A.K. Zvezdin, A. Kirilyuk, Th. Rasing, and A. V. Kimel, Anomalously Damped Heat-Assisted Route for Precessional Magnetization Reversal in an Iron Garnet, Phys. Rev. Lett. **122**, 027202 (2019).

[3] A. Brataas, A.D. Kent and H. Ohno, Current-induced torques in magnetic materials, Nat. Mater. **11**, 372 (2012).

[4] A.J. Schellekens, K.C. Kuiper, R.R.J.C. de Wit, and B. Koopmans, Ultrafast spin-transfer torque driven by femtosecond pulsed-laser excitation, Nat. Commun. **5**, 4333 (2014).

[5] M. Buess, T. P. J. Knowles, U. Ramsperger, D. Pescia, and C. H. Back, Phase-resolved pulsed precessional motion at a Schottky barrier, Phys. Rev. B **69**, 174422 (2004).

[6] H. Yuan, H. Gao, Y. Gong, J. Lu, X. Zhang, J. Zhao, Y. Ren, H. Zhao, and L. Chen, Photoinduced Spin Precession in Fe/GaAs(001) Heterostructure with Low Power Excitation, Appl. Phys. Express **6**, 073008 (2013).

[7] V. Saidl, P. Nemec, P. Wadley, K.W. Edmonds, R.P. Campion, V. Novak, B.L. Gallagher, F. Trojanek, and T. Jungwirth, Investigation of exchange coupled bilayer Fe/CuMnAs by pump–probe experiment, phys. stat. sol. RRL **11**, 1600441 (2017).

[8] G.-M. Choi, A. Schleife, D.G. Cahill, Optical-helicity-driven magnetization dynamics in metallic ferromagnets, Nat. Commun. **8**, 15085 (2017)

[9] L.Q. Shen, L.F. Zhou, J.Y. Shi, M. Tang, Z. Zheng, D. Wu, S.M. Zhou, L.Y. Chen, and H.B. Zhao, Dominant role of inverse Cotton-Mouton effect in ultrafast stimulation of magnetization precession in undoped yttrium iron garnet films by 400-nm laser pulses, Phys. Rev. B **97**, 224430 (2018).

[10] Y. Acremann, M. Buess, C. H. Back, M. Dumm, G. Bayreuther, and D. Pescia, Ultrafast generation of magnetic fields in a Schottky diode, Nature **414**, 51 (2001).

[11] G. Woltersdorf, M. Buess, B. Heinrich, and C.H. Back, Time Resolved Magnetization Dynamics of Ultrathin Fe(001) Films: Spin-Pumping and Two-Magnon Scattering, Phys. Rev. Lett. **95**, 037401 (2005).

[12] S. Qiao, J. Liu, G. Yan, J. Zhao, X. Zhang, S. Wang, and G. Fu, Magnetic field-modulated photo-thermo-electric effect in Fe/GaAs film, Appl. Phys. Lett. **107**, 182402 (2015).

[13] M. van Kampen, C. Jozsa, J. T. Kohlhepp, P. LeClair, L. Lagae, W. J. M. de Jonge, and B. Koopmans, All-Optical Probe of Coherent Spin Waves, Phys. Rev. Lett. **88**, 227201 (2002).

[14] N. Tesařová, P. Němec, E. Rozkotová, J. Zemen, T. Janda, D. Butkovičová, F. Trojánek, K. Olejník, V. Novák, P. Malý, and T. Jungwirth, Experimental observation of the optical spin-orbit torque, Nat. Phot. **7**, 492 (2013).

[15] J. Ryu, S. Lee, K.-J. Lee, and B.-G. Park, Current-Induced Spin–Orbit Torques for Spintronic Applications, Adv. Mater. **32**, 1907148 (2020).

[16] Gambardella, P., Miron, I. M., Current-induced spin-orbit torques, Phil. Trans. R. Soc. A **369**, 3175–3197 (2011).

[17] L. Chen, M. Decker, M. Kronseder, R. Islinger, M. Gmitra, D. Schuh, D. Bougeard, J. Fabian, D. Weiss1, and C.H. Back, Robust spin-orbit torque and spin-galvanic effect at the Fe/GaAs (001) interface at room temperature, Nat. Commun. **7**, 13802 (2016).

[18] K. Jhuria, J. Hohlfeld, A. Pattabi, E. Martin, A.Y.A. Cordova, X. Shi, R.L. Conte, S. Petit-Watelot, J.C. Rojas-Sanchez, G. Malinowski, S. Mangin, A. Lemaitre, M. Hehn, J. Bokor, R.B. Wilson, and J. Gorchon, Spin–orbit torque switching of a ferromagnet with picosecond electrical pulses, Nat. Electron. **3**, 680 (2020).

[19] M. I. Dyakonov and V. I. Perel, in *Optical Orientation*, edited by F. Meyer and B. P. Zakharchenya (North-Holland, 1984), pp. 11–71.

[20] M. Surýnek, L. Nádvorník, E. Schmoranzerová, and P. Němec, Quasi-nondegenerate pump–probe magnetooptical experiment in GaAs/AlGaAs heterostructure based on spectral filtration, New. J. Phys. **22**, 093065 (2020).

[21] D. J. Hilton and C. L. Tang, Optical Orientation and Femtosecond Relaxation of Spin-Polarized Holes in GaAs, Phys. Rev. Lett. **89**, 146601 (2002).

[22] J.M. Kikkawa and D.D. Awschalom, Resonant Spin Amplification in n-Type GaAs, Phys. Rev. Lett. **80**, 4313 (1998).

[23] D. Sprinzl, P. Horodyská, N. Tesařová, E. Rozkotová, E. Belas, R. Grill, P. Malý, and P. Němec, Influence of n-type doping on electron spin dephasing in CdTe, Phys. Rev. B **82**, 153201 (2010).

[24] P. Němec, E. Rozkotová, N. Tesařová, F. Trojánek, E. De Ranieri, K. Olejník, J. Zemen, V. Novák, M. Cukr, P. Malý, and T. Jungwirth, Experimental observation of the optical spin transfer torque, Nat. Phys. **8**, 411 (2012).

[25] D.H. Auston, Picosecond optoelectronic switching and gating in silicon, Appl. Phys. Lett. **26**, 101 (1975).

[26] B. Vermeersch, G. Pernot, H. Lu, J.-H. Bahk, A. Gossard, and A. Shakouri, Picosecond Joule heating in photoconductive switch electrodes, Phys. Rev. B **88**, 214302 (2013).

[27] W.K. Hiebert, A. Stankiewicz, and M. R. Freeman, Direct Observation of Magnetic Relaxation in a Small Permalloy Disk by Time-Resolved Scanning Kerr Microscopy, Phys. Rev. Lett. **79**, 1134 (1997).

[28] Th. Gerrits, H.A.M. van den Berg, J. Hohlfeld, L. Bär, and Th. Rasing, Ultrafast precessional magnetization reversal by picosecond magnetic field pulse shaping, Nature **418**, 509 (2002).

[29] Z. Wang, M. Pietz, J. Walowski, A. Förster, M. I. Lepsa, and M. Münzenberg, Spin dynamics triggered by subterahertz magnetic field pulses, J. Appl. Phys. **103**, 123905 (2008).

[30] K.-H. Ko, G.-M. Choi, Optical method of determining the spin diffusion length of ferromagnetic metals, J. Mag. Mag. Mater. **510**, 166945 (2020).

[31] W. Zawadzki, P. Pfeffer, R. Bratschitsch, Z. Chen, S. T. Cundiff, B. N. Murdin, and C. R. Pidgeon, Temperature dependence of the electron spin g-factor in GaAs, Phys. Rev. B **78**, 245203 (2008).

[32] T. Janda, P.E. Roy, R.M. Otxoa, Z. Šobáň, A. Ramsay, A.C. Irvine, F. Trojanek, M. Surýnek, R.P. Campion, B.L. Gallagher, P. Němec, T. Jungwirth, J. Wunderlich, Inertial displacement of a domain wall excited by ultra-short circularly polarized laser pulses, Nat. Commun. **8**, 15226 (2017).

[33] G.-M. Choi, J.H. Oh, D.-K. Lee, S.-W. Lee, K.W. Kim, M. Lim, B.-Ch. Min, K.-J. Lee, and H.-W. Lee, Optical spin-orbit torque in heavy metal-ferromagnet heterostructures, Nat. Commun. **11**, 1482 (2020).

[34] Ando, K. *et al.* Electrically tunable spin injector free from the impedance mismatch problem. *Nat. Mater.* **10**, 655–659 (2011).

[35] S. McPhail, C. M. Gürtler, F. Montaigne, Y. B. Xu, M. Tselepi, and J. A. C. Bland, Interface bonding versus strain-induced magnetic anisotropy in epitaxial Fe/semiconductor structures, Phys. Rev. B **67**, 024409 (2003).

[36] G. Wastlbauer and J. A. C. Bland, Structural and magnetic properties of ultrathin epitaxial Fe films on GaAs(001) and related semiconductor substrates, Advances in Physics **54**, 137 (2005).

[37] N. Tesařová, D. Butkovičová, R. P. Campion, A. W. Rushforth, K. W. Edmonds, P. Wadley, B. L. Gallagher, E. Schmoranzerová, F. Trojánek, P. Malý, P. Motloch, V. Novák, T. Jungwirth, and P. Němec, Comparison of micromagnetic parameters of the ferromagnetic semiconductors (Ga,Mn)(As,P) and (Ga,Mn)As, Phys. Rev. B **90**, 155203 (2014).

[38] T. F. Carruthers and J. F. Weller, Picosecond optical mixing in fast photodetectors, Appl. Phys. Lett. **48**, 460 (1986).

[39] R. H. Jacobsen, K. Birkelund, T. Holst, P. Uhd Jepsen, and S. R. Keiding, Interpretation of photocurrent correlation measurements used for ultrafast photoconductive switch characterization, J. Appl. Phys. **79**, 2649 (1996).

[40] S. D. Brorson, J. Zhang, and S.R. Keiding, Ultrafast carrier trapping and slow recombination in ion-bombarded silicon on sapphire measured via THz spectroscopy, Appl. Phys. Lett. **64**, 2385 (1994).

[41] G. E. Stillman, C. M. Wolfe, and J. O. Dimmock, Hall coefficient factor for polar mode scattering in n-type GaAs, J. Phys. Chem. Solids **31**, 1199 (1970).

[42] M. L. Lovejoy, M. R. Melloch, and M. S. Lundstrom, Temperature dependence of minority and majority carrier mobilities in degenerately doped GaAs, Appl. Phys. Lett. **67**, 1101 (1995).

[43] Z. Mics, A. D'Angio, S. A. Jensen, M. Bonn, and D. Turchinovich, Density-dependent electron scattering in photoexcited GaAs in strongly diffusive regime. Appl. Phys. Lett. **102**, 231120 (2013).

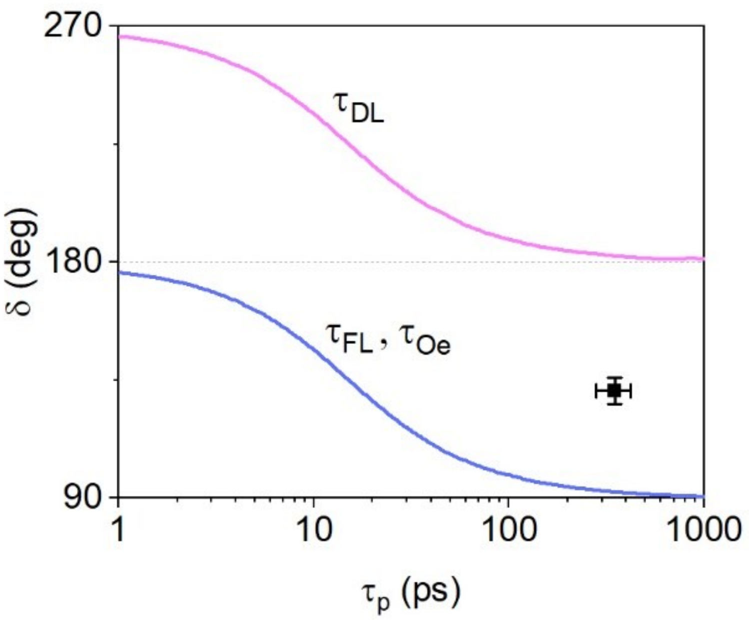

Fig. S.1: Dependence of magnetization precession phase on the photocurrent pulse duration. The lines depict magnetization precession phase δ deduced from simulated precessional dynamics triggered by Oersted-field pulses (τ_{Oe}) and purely field-like (τ_{FL}) and damping-like (τ_{DL}) SOT pulses of different duration (τ_p). Point: The experimentally measured phase and pulse duration corresponding to the experimental conditions of Fig. 2. A mixture of the damping-like and field-like SOTs is necessary to explain the observations.

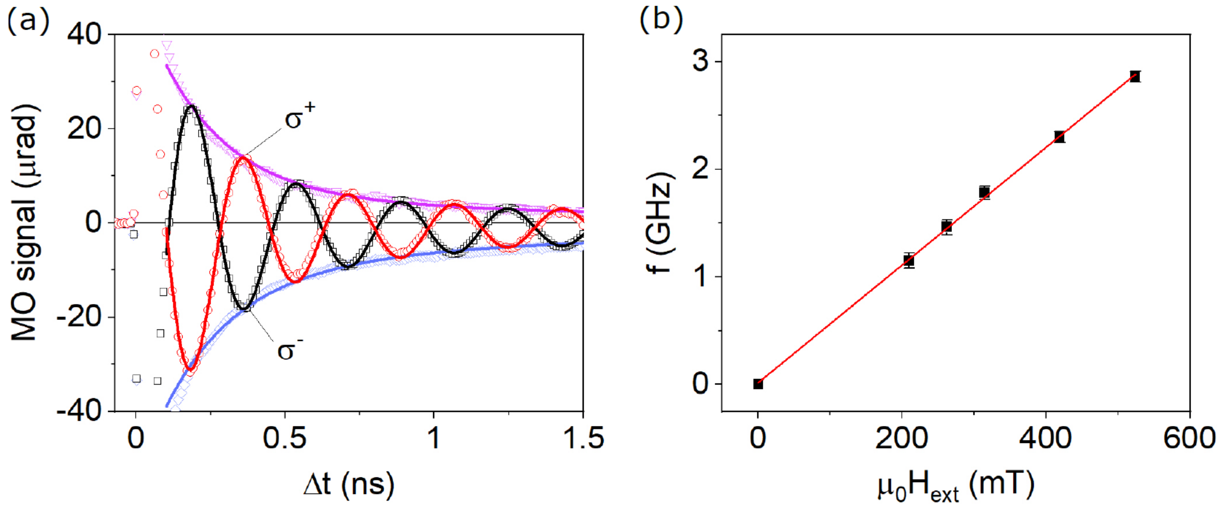

Fig. S.2: Electron spin dynamics in the Fe/GaAs PIN-diode structure. (a) Precession of the photo-injected spin-polarized electrons measured in perpendicularly applied external magnetic field of 500 mT (oscillatory signals) and without the field (curves with monotonous decays) for σ^+ and σ^- circularly-polarized laser pulses (points). The lines are fits by a double-exponential decay function with time constants $t_1 = 240 \pm 10$ ps and T_2^*=1470 ± 90 ps. **(b)** Electron Larmor precession frequency as a function of the external magnetic field (points). The line shows the fitted linear dependence with electron g-factor of 0.39 ± 0.02.

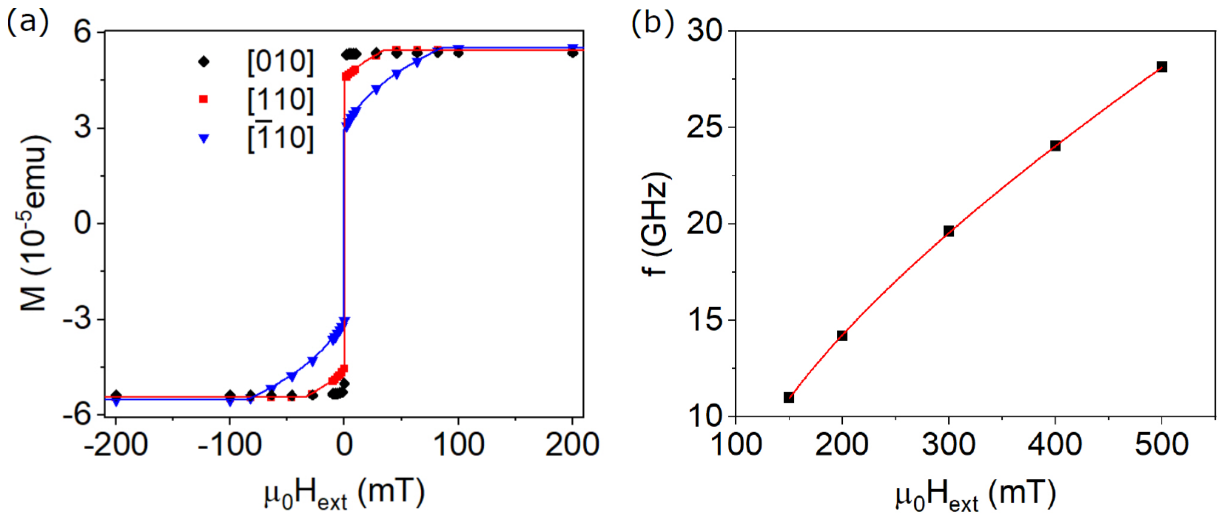

Fig. S.3: Magnetic characterization of the Fe/GaAs epilayer. (a) Magnetization components measured by SQUID magnetometry along different crystallographic directions. **(b)** Dependence of magnetization precession frequency f measured in a pump-probe MO experiment as a function of external magnetic field H_{ext} applied along the $[\bar{1}10]$ direction (points). The line is a fit by Eq. (S5).

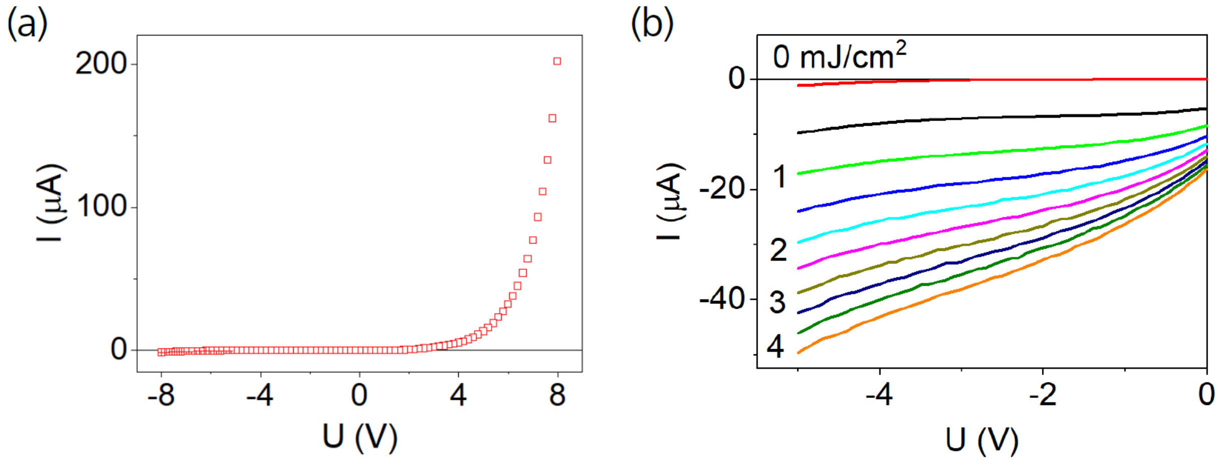

Fig. S.4: Electrical characterization of the Fe/GaAs photodiode. (a) V-I characteristics of the photodiode in darkness showing the ability to withstand large biases of both polarities. **(b)** V-I characteristics under illumination with a single fs-laser beam showing a nonlinear dependence of the photocurrent on the laser fluence.

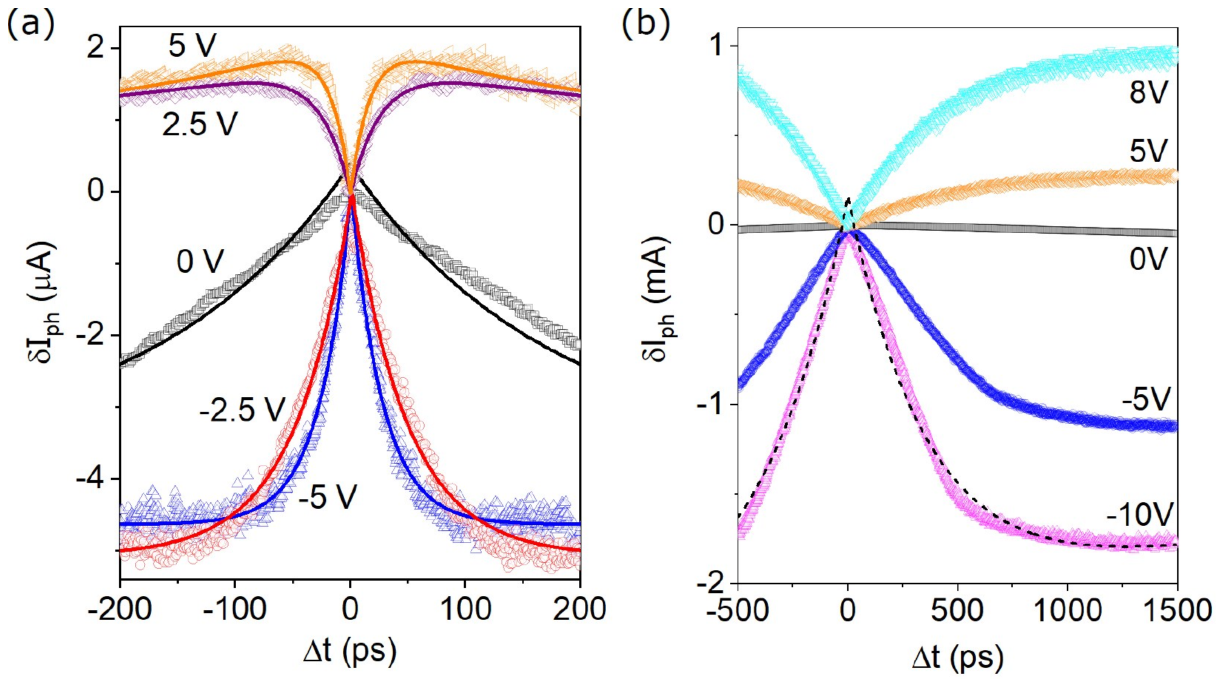

Fig. S.5: Characterization of ultrashort photocurrent pulses by photocurrent correlation technique. (a) Correlation measurements performed with ≈ 1 µm-wide laser spots. The time-averaged photocurrent I_{ph} is measured as a function of the mutual time delay Δt between two identical laser pulses for different applied biases. The change of photocurrent relative to its value at zero time delay, $\delta I_{ph} = I_{ph} - I_{ph}$ (Δt=0), is plotted. The measured data (points) are fitted (lines) by a single- and double-exponential model for reverse and forward biases, respectively. (b) Analogous measurements performed with ≈ 25 µm-wide laser spots (points) showing substantially longer current pulses due to more pronounced electric screening effects. Dashed line: fit by a single-exponential model.

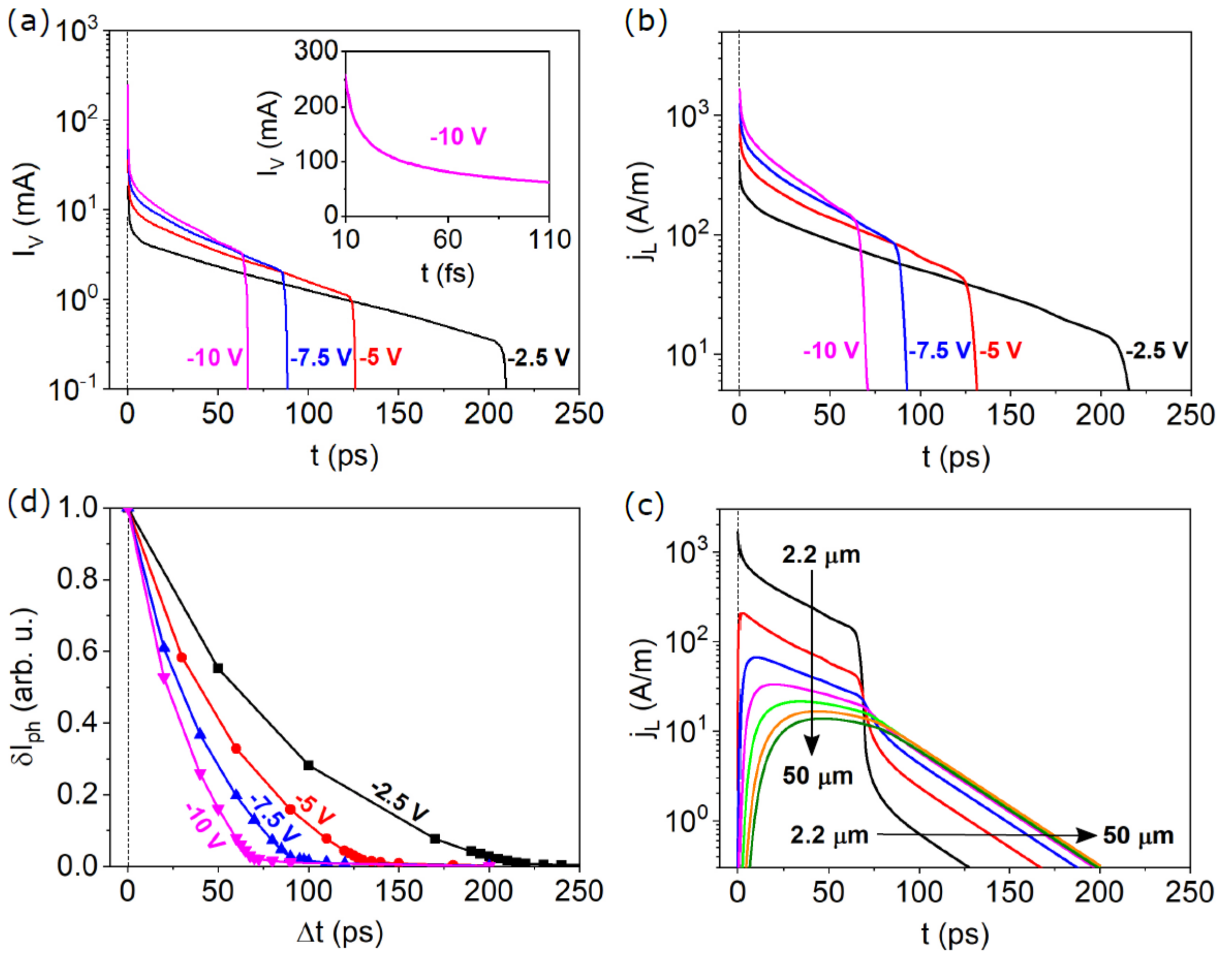

Fig. S.6: Numerical simulations of the charge transport dynamics. For different reverse voltage biases we show time evolutions of: (a) vertical current entering the *n*-electrode, (b) lateral current density flowing in the *n*-electrode at the edge of the illuminated area, and (d) corresponding normalized photocurrent correlation curves. The inset in part (a) depicts the very rapid initial dynamics of vertical current for -10 V, assuming that charge carriers are photoinjected by a δ-pulse (see Supplementary Note 7 for details). (c) Lateral current density time profiles at different distances (2.2, 8, 16, 25, 33, 42, and 50 μm) from the center of the illuminated spot for the applied voltage of -10 V. Note that y-scales in parts (a)-(c) are shown in logarithmic scale. As we discuss in Supplementary Note 7, to highlight the principal application potential of our device structure, we assumed in our simulations that charge carries are photoinjected by a δ-pulse. In reality, the excitation laser pulse has a Gaussian time profile with a FWHM of 100 fs and, consequently, the realistic current time profiles are convolutions of the laser pulse and the profiles shown in this Figure.

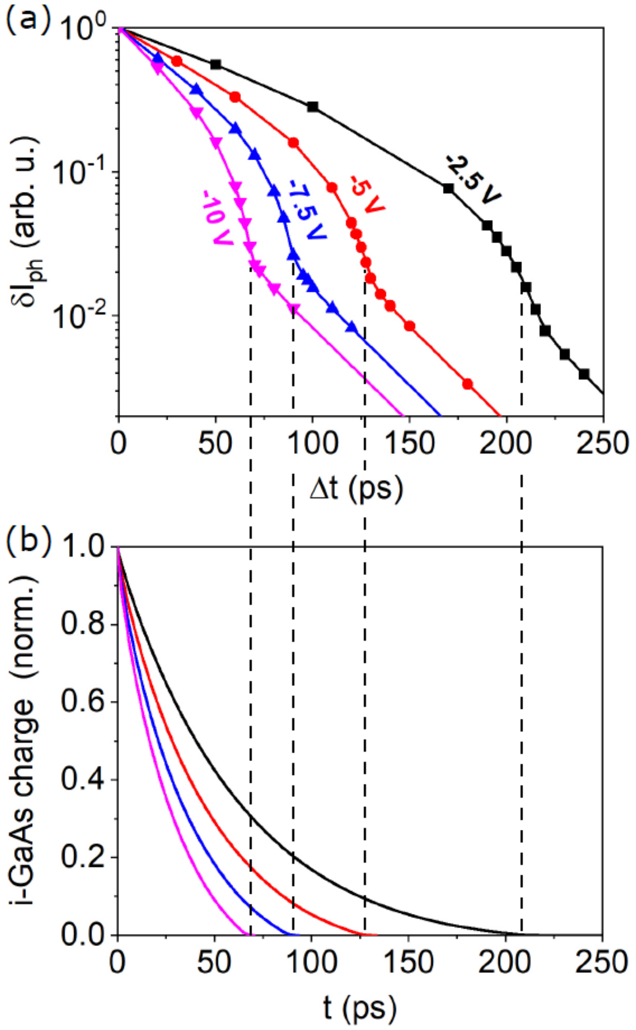

Fig. S.7: Demonstration of a dominant role of the *i*-GaAs layer discharging on the photocurrent correlation measurement in the Fe/GaAs *p*-*i*-*n* diode. (a) Data from Fig. S.6(d) re-plotted in logarithmic scale. (b) Charge depletion from *i*-GaAs for several applied biases.
